# Genetic diversity of *Mycobacterium tuberculosis* strains circulating in Botswana

**DOI:** 10.1371/journal.pone.0216306

**Published:** 2019-05-07

**Authors:** Tuelo Mogashoa, Pinkie Melamu, Serej D. Ley, Elizabeth M. Streicher, Thato Iketleng, Nametso Kelentse, Lucy Mupfumi, Margaret Mokomane, Botshelo Kgwaadira, Vladimir Novitsky, Ishmael Kasvosve, Sikhulile Moyo, Robin M. Warren, Simani Gaseitsiwe

**Affiliations:** 1 Department of Medical Laboratory Sciences, Faculty of Health Sciences, University of Botswana, Gaborone, Botswana; 2 Botswana Harvard AIDS Institute Partnership, Gaborone, Botswana; 3 DST-NRF Centre of Excellence in Biomedical Tuberculosis Research, South African Medical Research Council Centre for Tuberculosis Research, Division of Molecular Biology and Human Genetics, Faculty of Medicine and Health Sciences, Stellenbosch University, Cape Town, South Africa; 4 Swiss Tropical and Public Health Institute, Basel, Switzerland; 5 College of Health Sciences, School of Laboratory Medicine and Medical Sciences, University of KwaZulu-Natal, Durban, South Africa; 6 National Tuberculosis Reference Laboratory, Ministry of Health and Wellness, Gaborone, Botswana; 7 Harvard T.H. Chan School of Public Health, Boston, Massachusetts, United States of America; Universidad Nacional de la Plata, ARGENTINA

## Abstract

**Background:**

Molecular typing of *Mycobacterium tuberculosi*s (*M*.*tb)* isolates can inform Tuberculosis (TB) control programs on the relative proportion of transmission driving the TB epidemic. There is limited data on the *M*. *tb* genotypes that are circulating in Botswana. The aim of this study was to generate baseline data on the genetic diversity of *M*.*tb* isolates circulating in the country.

**Methods:**

A total of 461 *M*.*tb* isolates received at the Botswana National Tuberculosis Reference Laboratory between March 2012 and October 2013 were included in this study. Drug susceptibility testing was conducted using the BD BACTEC MGIT 960 System. *M*.*tb* strains were genotyped using spoligotyping and spoligotype patterns were compared with existing patterns in the SITVIT Web database. A subset of drug resistant isolates which formed spoligo clusters (n = 65) was additionally genotyped with 12-loci MIRU. Factors associated with drug resistance and clustering were evaluated using logistic regression.

**Results:**

Of the 461 isolates genotyped, 458 showed 108 distinct spoligotype patterns. The predominant *M*.*tb* lineages were Lineage 4 (81.9%), Lineage 2 (9%) and Lineage 1 (7.2%). The predominant spoligotype families within Lineage 4 were LAM (33%), S (14%), T (16%), X (16%). Three hundred and ninety-two (86%) isolates could be grouped into 44 clusters (2–46 isolates per cluster); giving a clustering rate of 76%. We identified 173 (37.8%) drug resistant isolates, 48 (10.5%) of these were multi-drug resistant. MIRU typing of the drug resistant isolates allowed grouping of 46 isolates into 14 clusters, giving a clustering rate of 49.2%. There was no association between age, sex, treatment category, region and clustering.

**Conclusions:**

This study highlights the complexity of the TB epidemic in Botswana with multiple strains contributing to disease and provides baseline data on the population structure of *M*.*tb* strains in Botswana.

## Introduction

Tuberculosis (TB) is amongst the top 10 causes of death throughout the world [1]. In 2017, an estimated 10.4 million people fell ill with TB; an estimated 1.3 million HIV uninfected people and 374 000 HIV infected people died of the disease. Botswana is a high TB burden country with an estimated TB incidence rate of 300 per 100,000 population [2]. According to the 2014 National TB report, about 60% of TB patients in Botswana are also co-infected with HIV [3]. Despite this high TB burden, studies to identify drivers of the TB epidemic in Botswana are sparse. The genetic diversity of *Mycobacterium tuberculosis* (*M*.*tb*) strains in Botswana remains largely unknown.

Molecular typing of *M*.*tb* is a useful tool to better understand the epidemiology of TB [4, 5]. Spacer oligonucleotide typing (spoligotyping), a genotyping method based on the amplification of the direct repeat (DR) region of the *M*.*tb* genome, is a rapid method to detect and differentiate *M*.*tb* clinical specimens into different lineages and sub-lineages [6, 7]. Mycobacterial Interspersed Repetitive Units—Variable Number of Tandem Repeats (MIRU-VNTR) is another polymerase chain reaction (PCR) based genotyping method used to differentiate *M*.*tb* strains[8]. Both, spoligotyping and MIRU-VNTR typing methods are widely used and results of these assays are stored in international databases [9, 10]. Another genotyping method, which has been proven useful in investigating TB transmission in the past, is the insertion sequence *6110* restriction fragment length polymorphism (IS*6110*-RFLP) typing [5, 11–14]. These assays enable the study of the global distribution and phylogenetic analyses of *M*.*tb* lineages across countries. *M*.*tb* lineages have been found to be distributed differently across the globe and thus reflect migration patterns and local epidemiology[15]. Tracking recent transmission chains in a TB endemic area enables the TB control programs to identify transmission hotspots and to implement effective targeted interventions [16].

Monitoring circulating *M*.*tb* strains remains a critical part of any TB control program as it can help improve our understanding of transmission dynamics in specific geographical locations [17]. Studies were previously conducted in Botswana by Lockman *et al*. to investigate the molecular epidemiology of *M*.*tb*. The findings of these studies in four communities and the population at large showed that there was IS*6110*-RFLP clustering rate of 25% and 38% respectively [18, 19]. The high IS*6110*-RFLP clustering rates were suggestive of active transmission within communities in Botswana. However, the studies conducted by Lockman *et al*. did not provide any detailed information on the phylogenetic lineages of the *M*.*tb* strains that were genotyped. Importantly, these studies were carried out more than 18 years ago so there is a need to generate more recent data on the *M*.*tb* diversity in Botswana. We generated more recent baseline data on the genetic diversity and distribution of circulating *M*.*tb* strains from specimens collected throughout Botswana.

## Methods

### Study setting and population

This was a retrospective, cross-sectional study. The study was approved by the University of Botswana Ethics Institutional Review Board and the Ministry of Health and Wellness Human Research Development Division. Permission was granted to use de-identified patient clinical information therefore patient consent was waived [Reference No: HPDME: 13/18/1 Vol. XI (140)]. We obtained all *M*.*tb* isolates (n = 461) that were available from the Botswana National Tuberculosis Reference Laboratory (NTRL) bio-repository. NTRL is a reference laboratory that receives sputum specimens for microscopy, culture and drug susceptibility testing (DST) from all public health facilities throughout the country. *M*.*tb* isolates included in this study were obtained from pulmonary and extrapulmonary samples received at NTRL from March 2012 to October 2013. These specimens were collected from 146 regional health posts, clinics and hospitals across all the 24 health districts and later sent to NTRL for culture. At the time of the study the indications for culture were as follows: HIV positive patients with 2 negative sputum smear results, new patients who are smear positive at month 3 of treatment, all retreatment patients, patients who have received anti-tuberculosis therapy (ATT) for more than 1 month in the past, all children, patients who develop TB during or after isoniazid preventative (IPT), symptomatic individuals who are at higher risk of MDR-TB such as health care workers, MDR-TB contacts and laboratory personnel, patients with suspected cryptogenic TB as well as fluids or tissues which are suspected to be infected by *M*.*tb*[3]. The patients’ clinical information was obtained from the laboratory patient management system.

The administrative health districts were grouped into 4 regions namely; North West, Central, Southern and South West. A central location was chosen for each region and its coordinates were used to plot the distribution of lineages in each region. The Botswana map was generated by using freely available R packages ggmap, maps and mapdata. The maps with pie charts were edited using Adobe Illustrator CS6. The get_map() is a smart wrapper that queries Stamen Maps by specified longitude (19.8, 29) and latitude (-18, -26.7).

### Drug susceptibility testing

Drug susceptibility testing (DST) for the first line drugs; isoniazid (INH), rifampicin (RIF), ethambutol (EMB) and streptomycin (STR) was performed using the BACTEC MGIT 960 System (Becton Dickinson Diagnostic Systems, Sparks, MD, USA) following the manufacturer’s instructions. Susceptibility to first line drugs was tested at the following concentrations: 0.1 μg/mL and 0.4 μg/mL for low and high concentrations of isoniazid respectively, 1.0 μg/mL for rifampicin and streptomycin and 5.0 μg/mL for ethambutol. Isolates were categorized into groups based on the resistance profile. Resistance to only one drug was termed monoresistance, resistance to at least isoniazid (INH) and rifampicin (RIF) were termed multi-drug resistance (MDR) and resistance to more than one first line drug but not meeting MDR criteria was termed polyresistance.

### DNA extraction

DNA was extracted from the BD MGIT cultures (BD Biosciences, Sparks, MD, USA) using the GenoLyse DNA extraction kit version 1.0 (Hain LifeScience, GmBH, Nehren, Germany) following the manufacturer’s instructions.

### Spoligotyping

Spoligotyping was performed as previously described by Kamerbeek *et al*. [6] on 461 isolates. A commercially available spoligotyping kit (Mapmygenome, Hyderabad India) was used for amplification, hybridization and detection of DNA. The direct repeat (DR) region of the isolates was amplified by PCR using oligonucleotides primers DRa (5’-GGTTTTGGGTCTGACGAC-3’) biotinylated at the 5’ end) and DRb (5’-CCGAGAGGGGACGGAAAC-3’) derived from the DR sequence for 30 cycles at a Tm of 55°C. The characterized *M*.*tb* strains H37Rv (laboratory reference strain) and *Mycobacterium bovis* (*M*.*bovis)*- Bacillus Calmette-Guérin (BCG) were included as positive controls; distilled water with PCR mix was used as negative control in each run. The resulting spoligotype patterns were reported in octal and binary formats and compared to existing patterns in the international genotyping database SITVIT2 [10] available at the following address: http://www.pasteur-guadeloupe.fr:8081/SITVIT_ONLINE/. Spoligotype patterns were grouped as spoligotype international types (SITs) if they shared identical patterns with existing patterns in the database. Isolates that did not share their patterns with previously reported patterns were termed “unknown”. *M*.*tb* lineages and families were assigned based on spoligotyping data submitted to the SITVIT2 database. A clustering rate for spoligotyping was calculated using the formula (nc−c)/n, where n_c_ is the total number of isolates clustered by spoligotyping, c is the number of clusters and n is the total number of isolates that were genotyped. A cluster was defined as two or more isolates with identical spoligotype patterns.

### MIRU typing

To enhance the discriminatory resolution to identify possible clusters of transmission we applied the standardized 12-loci MIRU-VNTR protocol by Supply *et al*. [20] to the subset of isolates that were resistant to either INH or RIF or to both INH and RIF and which clustered according to spoligotyping (n = 65). *M*.*tb* H37Rv DNA was used as positive control and DNA-free water as a negative control. The PCR products were fractionated by electrophoresis in 2% agarose gels in 1XTris/Borate/Ethylenediaminetetraacetic acid buffer (TBE buffer) at 65V for 16hrs to allow for clear discrimination. The number of repeats at each locus was calculated from the size of DNA fragments according to the standardized table (http://www.MIRU-VNTRplus.org). The results were recorded in a digital format where each digit represented the number of repeats at a particular locus. A clustering rate for MIRU was calculated using the formula (nc−c)/n, where n_c_ is the total number of isolates clustered by MIRU, c is the number of clusters and n is the total number of MIRU typed isolates in the study. A cluster was defined as two or more isolates with identical MIRU patterns.

### Data analysis

STATA version 14 (Stata Corp, College Station, TX, USA) was used for statistical analysis. Factors associated with drug resistance and clustering were evaluated using univariate logistic regression. A p-value of <0.05 was considered statistically significant.

## Results

**Fig 1:**
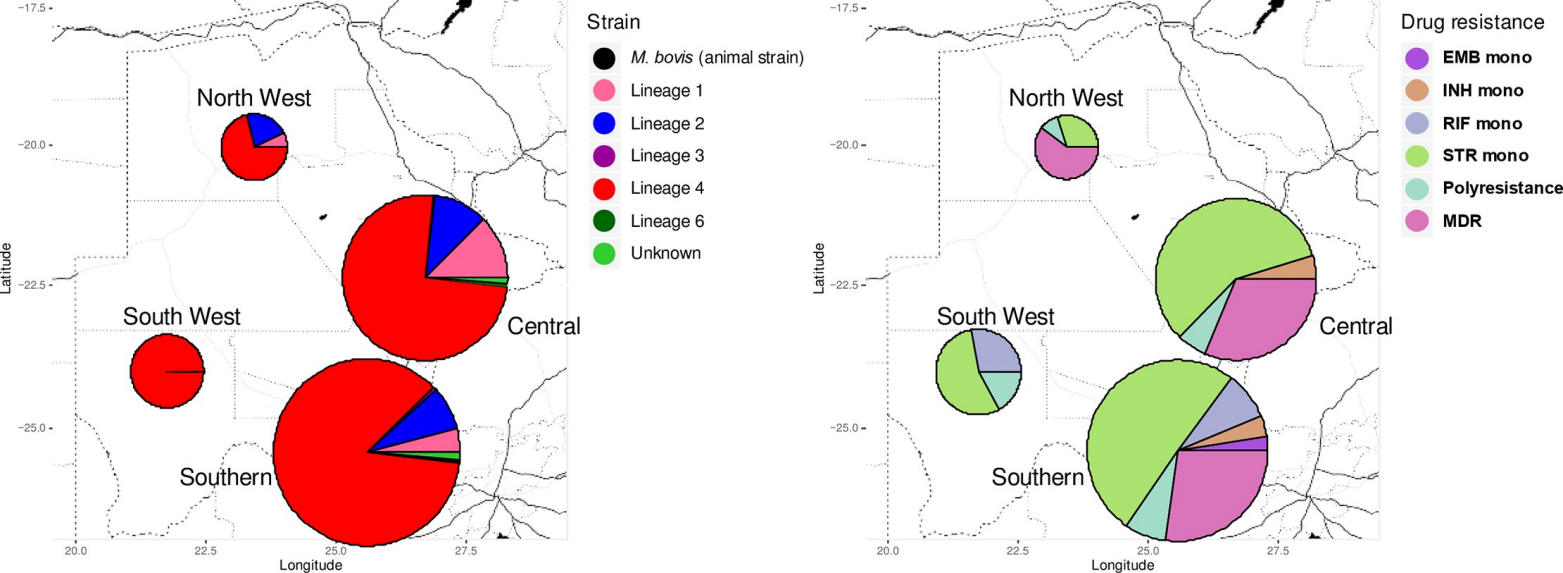
Geographical distribution of *M*.*tb* lineages and drug resistance profiles in Botswana. A map of Botswana is shown in which pie charts indicate (A) the *M*.*tb* lineages (see key). On the right map (B) the pie charts indicate the drug resistance profiles of the isolates (see key). The size of the pie chart is proportional to the number of isolates in the region.

A total of 458/461 isolates were successfully spoligotyped. Of these 458 isolates, 174 (38%) were from the Central region, 222 (48.5%) Southern, 34 (7.4%) South West and 28 (6.1%) were from the North West region (Fig 1). The TB patients had a median age of 36 [IQR 27–44], with most of them (47%) falling in the age group 20–39 years. Treatment category I (new cases) made up the largest proportion of our cases (43%) compared to retreatment and MDR categories. Among the 334 TB patients whose HIV status was known, 239 (72%) were HIV positive, 95 (28%) HIV negative (Table 1).

**Table 1:** Baseline demographics of the patients in the study.

Characteristic	n = 458	
	n	%
**Sex (n = 454)**		
**Male**	232	50.7
**Female**	225	49.1
**Unknown**	1	0.2
**Age, median [IQR]**	**36, [44–27]**	
**<20 years**	55	12.9
**20–39 years**	214	46.7
**40–59 years**	124	27.1
**>60 years**	33	7.2
**Unknown**	32	7.0
**HIV status**		
**Positive**	239	52.2
**Negative**	95	20.7
**Unknown**	124	27.1
**Specimen type**		
**Extra-pulmonary**	38	8.3
**Pulmonary**	415	90.6
**Unknown**	5	1.1
**Smear result**		
**Smear negative**	123	26.9
**Smear positive**	335	73.1
**Treatment category at baseline**		
Category I*	196	42.7
Category II**	109	23.7
**Multi drug resistant TB (MDR)**	23	5.0
**Unknown**	130	28.4
**Region**		
**Central**	174	38.0
**South West**	34	7.4
**North West**	28	6.1
**Southern**	222	48.5

* Category 1: This includes new cases, contacts and children

**Category 2: This includes retreatment cases, relapse cases

Based on the phenotypic DST results, 268 isolates (58.5%) were susceptible to all four first line drugs while 173 isolates (37.8%) were resistant to at least one drug, 17 isolates (3.7%) did not have DST results. Among isolates with drug resistance, 111 (24.2%) were monoresistant, 48 (10.5%) were MDR while 14 (3.1%) were polyresistant (Table 2). Out of 173 patients whose isolates were resistant to at least one drug, 54% were males, 52% were HIV positive (127 patients had a known HIV status), 54% belonged to the 20–39 age group.

**Table 2:** Phenotypic drug resistance profiles and lineages of the *M*.*tb* isolates in the study.

Drug resistance profile ^1^	Lineage 1	Lineage 2	Lineage 4	Other^	Total
** **	(n = 33) n (%)	(n = 41) n (%)	(n = 375) n (%)	(n = 9) n (%)	(n = 458) n (%)
**#Monoresistant**	**-**	**1 (2.4)**	**110 (29.3)**	**-**	**111 (24.2)**
**INH**	-	1(2.4)	5 (1.3)	-	6 (1.3)
**RIF**	-	-	12 (3.2)	-	12 (2.6)
**STR**	-	-	91(24.3)	-	91 (19.9)
**EMB**	-	-	2 (0.5)	-	2 (0.4)
**Polyresistant	**1 (3.0)**	-	**13 (3.5)**	**-**	**14 (3.1)**
**INH + STR**	-	-	7 (1.9)	-	7 (1.5)
**INH +EMB**	-	-	3 (0.8)	-	3 (0.7)
**INH + STR + EMB**	1 (3.0)	-	1 (0.3)	-	2 (0.4)
**STR +EMB**	-	-	2 (0.5)	-	2 (0.4)
***MDR	**9 (27.2)**	**13 (31.7)**	**25 (6.7)**	**1 (11.1)**	**48 (10.5)**
**INH + RIF**	-	5 (12.2)	5 (1.3)	-	10 (2.2)
**INH + RIF + EMB**	1 (3.0)	3 (7.3)	6 (1.6)	-	10 (2.2)
**INH + RIF + STR**	-	1 (2.4)	2 (0.5)	-	3 (0.7)
**INH + RIF + STR + EMB**	8 (24.2)	4 (9.8)	12 (3.2)	1 (11.1)	25 (5.5)
**Pan-susceptible**	**21 (63.6)**	**23 (56.1)**	**217 (57.9)**	**7 (77.8)**	**268 (58.5)**
No DST result*	2 (6.1)	4 (9.8)	10 (2.7)	1 (11.1)	17 (3.7)

Drug resistance profile

^**1**^: INH- Isoniazid, RIF- rifampicin, STR- streptomycin, EMB- Ethambutol

#Monoresistant: resistant for one of the drugs: INH, EMB, RIF, STR

**Polyresistant: resistance to more than one first-line anti-TB drug, other than both INH and RIF (drug-resistant tuberculosis other than MDR-TB)

***MDR: Multi-drug resistance (resistance to at least both isoniazid and rifampicin

^Other includes isolates from an unknown Lineage

*First line DST was not done for these isolates

The *M*.*tb* lineages identified in our dataset belonged to Lineage 4 (Euro American) (81.9%), Lineage 2 (East Asian) (9.0%), Lineage 1 (Indo-Oceanic) (7.2%), and Lineage 3 (East-African-Indian) (0.4%).Five isolates (1.1%) could not be assigned to any of the known lineages. Within the Lineage 4 strains, spoligo families included Latin American Mediterranean (LAM) (32.8%) followed by X (16.4%), T (15.9%), S (13.5%), Haarlem (4.6%), U (0.7%) and *H37Rv* (0.4%). All Lineage 2 strains belonged to the Beijing family. Lineage 1 strains included the EAI (6.8%) and MANU (0.4%) families whereas Lineage 3 strains included the CAS family (0.4%). Only one case of *M*. *bovis* (0.2%) and one case of AFRI_1 (*M*. *africanum*, Lineage 6*)* (0.2%) were detected (Table 3).

**Table 3:** Proportion of *M*. *tuberculosis* lineages and Spoligotype defined families (n = 458).

**Lineage**	**Family**	**n (%)**
**Lineage 1 (Indo-Oceanic)**		**33 (7.2)**
	EAI	31 (6.8)
	MANU	2 (0.4)
**Lineage 2 (East-Asian)**	Beijing	**41 (9.0)**
**Lineage 3 (East-African-Indian)**	CAS	**2 (0.4)**
**Lineage 4 (Euro-American)**		**375 (81.9)**
	LAM	150 (32.8)
	Haarlem	21 (4.6)
	H37Rv	2 (0.4)
	S	62 (13.5)
	T	73 (15.9)
	U	3 (0.7)
	X	75 (16.4)
**Lineage 6 (West African 2)**	AFRI_1	**1 (0.2)**
**Animal strains (Bovis)**	Bovis2	**1 (0.2)**
**Unknown**	Unknown	**5 (1.1)**

We identified 108 distinct spoligotype patterns; 44 clusters (2–46 isolates per cluster) and 69 had unique spoligotype patterns (S1 Table). The clustering rate for spoligotyping was found to be 76%. Lineage 4 (Euro American) was also the most prevalent lineage within the drug resistant TB isolates (81%) (Table 4). Sixty-five of the 173 drug resistant isolates were successfully genotyped by MIRU. We identified 33 MIRU patterns among the drug resistant isolates; 46 isolates formed 14 clusters (2–6 isolates per cluster) and 19 isolates had unique MIRU patterns. The MIRU clustering rate for a subset of drug resistant isolates was calculated to be 49.2% (S2 Table). Using univariate logistic regression analysis there was no association between age, sex, HIV status, treatment category or geographical region and clustering (Table 4). The risk of resistance to at least one anti-TB drug was 1.4 (p = 0.067) and 1.1 (p = 0.676) times higher for females and HIV negative patients respectively, but this was not statistically significant. We also could not detect an association between sex, age, HIV status, *M*.*tb* lineage and drug resistance (Table 5)

**Table 4:** Univariate logistic regression analysis for the odds of clustering.

Characteristic	clustered isolates (n = 389)	unique isolates (n = 69)	OR (95%CI)	p values
**Age**	**n, (%)**			
<20 years*	47 (2.9)	8 (12.7)		
**20–39 years**	180 (49.6)	34 (54.0)	0.90 (0.39,2.08)	0.81
**40–59 years**	106 (29.2)	18 (28.6)	1.00 (0.41,2.47)	0.99
**>60 years**	30 (8.3)	3 (4.8)	1.70 (0.42,6.93)	0.46
**Sex**				
Female*	200 (51.4)	32 (46.4)		
**Male**	189 (48.6)	36 (52.2)	1.19 (0.71,1.99)	0.51
**Treatment category**				
Category I*	165 (42.4)	31 (44.9)		
**Category II**	97 (24.9)	12 (17.4)	1.52 (0.75,3.10)	0.25
**MDR**	22 (5.7)	1 (1.45)	4.13 (0.54,31.8)	0.17
**HIV status**				
Negative*	78 (20.1)	17 (24.6)		
**Positive**	204 (52.4)	35 (50.7)	1.27 (0.67,2.40)	0.46
**Region**				
Central*	152 (39.0)	22 (31.9)		
**North West**	22 (5.7)	6 (8.7)	0.53 (0.19,1.45)	0.22
**South West**	26 (6.7)	8 (11.6)	0.47 (0.19,1.17)	0.10
**Southern**	189 (48.6)	33 (47.8)	0.83 (0.46,1.48)	0.53

*Reference category.

**Table 5:** Logistic regression for factors associated with drug resistance.

** **	**Pan-susceptible** **n (%)**	**Any drug resistance** **n (%)**	**Association with resistance to at least 1 drug**	** **
**Variable**			OR (95%CI)	*p* value
**Sex**				
Male*	144 (53.7)	78 (45.1)		
**Female**	121 (45.2)	94 (54.3)	1.434 (0.976–2.108)	0.067
**Age**				
<20 years*	25 (10.1)	25 (15.5)		
**20–39 years**	133 (53.6)	78 (48.5)	0.586 (0.315–1.091)	0.092
**40–59 years**	70 (28.3)	48 (29.8)	0.686 (0.352–1.333)	0.266
**>60 years**	20 (8.1)	10 (6.2)	0.500 (0.195–1.280)	0.148
**Region**				
Central*	98 (36.6)	69 (39.8)		
**South West**	20 (7.5)	13 (7.5)	0.923 (0.430–1.980)	0.837
**North West**	15 (5.6)	10 (5.8)	0.947 (0.402–2.232)	0.901
**Southern**	135 (50.4)	81 (46.8)	0.852 (0.564–1.288)	0.448
**HIV Status**				
Positive*	141 (52.6)	91 (52.6)		
**Negative**	53 (19.8)	38 (22)	1.111 (0.676–1.819)	0.676
**Smear result**				
Positive*	196 (73.1)	133 (76.9)		
**Negative**	72 (26.9)	40 (23.1)	0.819 (0.525–1.277)	0.378
**Lineage**				
Lineage 2*	23 (8.6)	14 (8.1)		
**Lineage 3**	21 (7.8)	10 (5.8)	0.782 (0.286–2.136)	0.632
**Lineage 4**	217 (81)	148 (85.6)	1.120 (0.558–2.248)	0.749
**Other#**	7 (2.6)	1 (0.6)	0.235 (0.026–2.114)	0.196

* Reference category

# Other includes *M*.*tb* isolates which belong to Lineage 3, Lineage 6, Bovis (animal strains), Unknown

OR = Odds ratio

CI = Confidence interval

n = 441, 17 isolates excluded from analysis because they did not have DST result

## Discussion

This study provides most recent insights on the genetic diversity of *M*.*tb* strains circulating in Botswana. The analyzed *M*.*tb* strains belong to Lineages 1–4 and 6 with a predominance of Lineage 4 strains. Within Lineage 4 strains, the predominant families were LAM (Latin American Mediterranean), S, T and X. Previous studies have shown that the TB epidemic in Africa is dominated by Lineage 4 (Euro-American) strains [21]. In Southern Africa LAM11-ZWE and Lineage 2 (East Asian) have been shown to be dominant [21].

The overall strain diversity observed in Botswana is similar to that of *M*.*tb* lineages in neighboring countries South Africa, Zambia and Zimbabwe [21–23]. The presence of the AFRI_1 (Lineage 6) strain in the central region of Botswana is noteworthy. This *M*.*tb* family is usually not found outside of West African countries [24–26]. In a recent study by Mbugi *et al* to assess the genetic diversity of *M*.*tb* in Africa, countries that share borders with Botswana such as South Africa, Namibia, Zambia and Zimbabwe did not report any Lineage 6 strains. Lineage 6 strains were only reported in Cameroon, Nigeria and Sudan which is consistent with other published data [22]. The Lineage 6 *M*.*tb* strain detected in our study could have been introduced through recent human migrations between West African countries and Botswana. However, since no additional clinical or ethnicity data from the respective patient was available, we can only speculate about the origin of the strain and more investigations are required to assert this hypothesis.

An interesting finding in this study is the presence of an *M*. *bovis* strain*–*the causative agent of bovine tuberculosis (BTB) [23, 27]. The presence of only one animal strain in our dataset suggests that there is either a low burden of BTB among cattle or low transmission rates from cattle to humans. There is limited data on the prevalence of BTB in Botswana and in one study carried out in the Northern region of Botswana by Jori *et al*. in 2013 the prevalence of BTB was reported to be very low [27]. The impact of BTB as a zoonotic disease in Botswana remains to a greater extent unknown and warrants further studies. There were 2 (0.4%) isolates with a genotype of the reference strain H37Rv; reasons for this might be laboratory contamination of patient samples with the reference strain. This however could not be further investigated.

There is limited published data about *M*.*tb* genotype clustering rates in African countries. The clustering rate obtained in this study by spoligotyping was 76% and comparable with those previously reported by Mulenga *et al*. in a study at Ndola district in Zambia [13]. A previous study in Botswana by Lockman *et al*., obtained a population clustering rate of 38% by IS*6110* RFLP [18]. This clustering rate was much lower than that obtained by spoligotyping, which is to be expected since IS*6110* RFLP is a more discriminatory genotyping method. The clustering rate for a subset of drug resistant isolates was 49.2%. These results cannot be used to infer transmission since 12 loci MIRU is not discriminatory enough to identify possible transmission chains [16]. Compared to other studies conducted in Zambia and Benin showing MIRU clustering rates of 37.7% and 34% respectively, our clustering rate is higher than those obtained in both countries [13]. Further investigations on the transmission dynamics in Botswana through the use of highly discriminatory genotyping techniques such as whole genome sequencing are required to determine the proportion of clustered (i.e. transmitted) versus non-clustered (i.e. acquired/imported/reactivated) drug resistant TB cases. The presence of unclustered or unique isolates suggests that TB reactivation and/or individual cases of imported TB from neighbouring countries may be playing a role in local TB epidemiology [28]. Since the unclustered isolates are acquired or reactivated, diagnosis or treatment compliance could be a problem and may need to be investigated.

According to the previous national anti-tuberculosis drug resistance survey conducted between 2003 to 2013, there has been an increase in cases of drug resistant TB in Botswana; the proportion of new patients with any resistance to INH and RIF had increased 3.1-fold since 2002. In the TB drug resistance survey conducted between 20017 and 2008, 82 out of the 924 (8.9%) isolates which were included in the survey were resistant to either INH, RIF or EMB [29]. Among new patients (n = 70) 7.6% were resistant against INH, (33) 3.6% were resistant against RIF, (17) 1.8% were resistant against EMB and (96) 10.6% were resistant against streptomycin [30]. In this study, there were 196 (43%) new TB cases. Almost 40% of the isolates were resistant to at least one first line anti-TB drug, with monoresistance detected mainly to streptomycin (19.9%) and rifampicin (2.6%). This however, does not reflect the drug resistance rates in the general population due to the national TB program’s criteria for culturing specimens at the time of the study. The high rates of streptomycin resistance could be attributed to the fact that streptomycin was at the time of the study used in empiric retreatment regimens before drug resistance testing results are available, it is also commonly used to treat other bacterial infections [29, 31]. The presence of both INH and RIF monoresistance indicates that there is an increased risk for development of MDR-TB especially in cases where patients are not properly adhering to treatment [32, 33]. There were no significant associations between drug resistance and sex, age, lineage or smear result in this study. Similar findings have been reported in other countries [34].

We acknowledge that our study had some limitations. Firstly, there is a selection bias in the study population because cultures were only performed for a certain group of patients who met the national culture inclusion criteria as described in the methods section. As a result, our findings may not be generalizable to the entire population. Secondly, the use of spoligotyping and 12-loci MIRU typing does not allow to draw conclusions about recent transmission of *M*.*tb* in Botswana. 15 loci or 24 loci-MIRU-VNTR typing methods with an automated system could potentially already reduce cluster rates, but despite having frequently been used for transmission analyses, a recent study by Meehan *et al*. showed that MIRU-typing generally is not discriminatory enough for that purpose [16]. MIRU typing may nevertheless serve as a first step in surveillance of potential transmission hotspots in a population, but further investigations using whole genome sequencing (WGS) are required to provide additional resolution [16]. As shown by other studies, WGS is able to discriminate between TB isolates at the single nucleotide level (SNP) which can in turn identify TB cases which are most likely to linked by transmission [35, 36]. This genotyping technique has improved understanding of the TB epidemic in other Southern African countries.

Thirdly, there are other variables than those analyzed that could have played a role in the dominance and distribution of *M*.*tb* lineages; these include the frequency of social gatherings, family history of TB and travel history that were not investigated in this study. Finally, we did not have treatment outcome data for the patients and could therefore not determine if there is any association between treatment outcomes and *M*.*tb* lineage. Despite these limitations, our study provides important baseline information on the circulating *M*.*tb* strains and on the types of *M*.*tb* drug resistance present in Botswana. Such data is invaluable for the planning of future studies investigating the molecular epidemiology of *M*.*tb* and its transmission dynamics.

## Conclusions

Our study provides baseline data on the genetic diversity of *M*.*tb* strains circulating in Botswana which could be used for future molecular epidemiology studies and contributes to the body of knowledge regarding global *M*.*tb* genetic diversity. It also shows the utility of spoligotyping and 12 loci MIRU in robustly assigning lineages and understanding the *M*.*tb* population structure in a TB endemic setting. However, there is a need for further epidemiological studies over extended period of time using whole genome sequencing to determine the precise genetic diversity and the transmission dynamics of *M*.*tb* strains in Botswana using a sample set that is representative of the entire country.

## Supporting information

S1 Table Spoligotype international types (SIT) and corresponding spoligotyping-defined families and lineages of *M. tuberculosis* isolates included in the study. (DOCX)

S2 Table MIRU types (MIT) and corresponding spoligotyping-defined families and lineages for some of the drug resistant *M. tb* isolates in the study. (DOCX)

## References

[1] WHO. The top 10 causes of death: WHO; 2018 [updated 24 May 2018; cited 2019 15 March]. Available from: https://www.who.int/news-room/fact-sheets/detail/the-top-10-causes-of-death.

[2] WHO. Global Tuberculosis Report WHO; 2018 [updated 28 February 2019; cited 2018 20 September]. Available from: https://www.who.int/tb/publications/global_report/en/.

[3] National Tuberculosis Programme Manual Gaborone: Botswana Ministry of Health; 2007 [cited 2019 5th January]. Sixth:[Available from: https://www.who.int/hiv/pub/guidelines/botswana_tb.pdf.

[4] Ereqat S, Nasereddin A, Azmi K, Abdeen Z, Greenblatt CL, Spigelman M, et al Genetic characterization of Mycobacterium tuberculosis in the West Bank, Palestinian Territories. BMC research notes. 2012;5(1):270 10.1186/1756-0500-5-270 22676404 PMC3441885

[5] Van Soolingen D, Hermans PWM, De Haas PEW, Soll DR, Van Embden JDA. Occurrence and stability of insertion sequences in Mycobacterium tuberculosis complex strains: Evaluation of an insertion sequence-dependent DNA polymorphism as a tool in the epidemiology of tuberculosis. Journal of Clinical Microbiology. 1991;29(11):2578–86. 1685494 10.1128/jcm.29.11.2578-2586.1991 PMC270376

[6] Kamerbeek J, Schouls L Fau—Kolk A, Kolk A Fau—van Agterveld M, van Agterveld M Fau—van Soolingen D, van Soolingen D Fau—Kuijper S, Kuijper S Fau—Bunschoten A, et al Simultaneous detection and strain differentiation of Mycobacterium tuberculosis for diagnosis and epidemiology. Journal of Clinical Microbiology. 1997;35(4):907–14. 9157152 10.1128/jcm.35.4.907-914.1997 PMC229700

[7] Streicher EM, Victor TC, van der Spuy G, Sola C, Rastogi N, van Helden PD, et al Spoligotype Signatures in the Mycobacterium tuberculosis Complex. Journal of Clinical Microbiology. 2007;45(1):237–40. PubMed Central PMCID: PMCPMC1828946. 10.1128/JCM.01429-06 17065260 PMC1828946

[8] Supply P, Lesjean S, Savine E, Kremer K, Van Soolingen D, Locht C. Automated high-throughput genotyping for study of global epidemiology of Mycobacterium tuberculosis based on mycobacterial interspersed repetitive units. Journal of Clinical Microbiology. 2001;39(10):3563–71. 10.1128/JCM.39.10.3563-3571.2001 11574573 PMC88389

[9] Allix-Béguec C, Harmsen D, Weniger T, Supply P, Niemann S. Evaluation and strategy for use of MIRU-VNTRplus, a multifunctional database for online analysis of genotyping data and phylogenetic identification of Mycobacterium tuberculosis complex isolates. Journal of Clinical Microbiology. 2008;46(8):2692–9. 10.1128/JCM.00540-08 18550737 PMC2519508

[10] Demay C, Liens B Fau—Burguiere T, Burguiere T Fau—Hill V, Hill V Fau—Couvin D, Couvin D Fau—Millet J, Millet J Fau—Mokrousov I, et al SITVITWEB—a publicly available international multimarker database for studying Mycobacterium tuberculosis genetic diversity and molecular epidemiology. Infect Genet Evol. 2012;12(4):755–66. Epub 2012 Feb 17. 10.1016/j.meegid.2012.02.004 22365971

[11] Barnes PF, Cave MD. Molecular Epidemiology of Tuberculosis. New England Journal of Medicine. 2003;349(12):1149–56. 10.1056/NEJMra021964 13679530

[12] Mbugi EV, Katale BZ, Siame KK, Keyyu JD, Kendall SL, Dockrell HM, et al Genetic diversity of Mycobacterium tuberculosis isolated from tuberculosis patients in the Serengeti ecosystem in Tanzania. Tuberculosis. 2015;95(2):170–8. 10.1016/j.tube.2014.11.006 25522841 PMC4364622

[13] Mulenga C, Shamputa IC, Mwakazanga D, Kapata N, Portaels F, Rigouts L. Diversity of Mycobacterium tuberculosis genotypes circulating in Ndola, Zambia. BMC Infect Dis. 2010;10(177). Epub 2010/06/23. 10.1186/1471-2334-10-177 20565802 PMC2906459

[14] van Embden JD, Cave MD, Crawford JT, Dale JW, Eisenach KD, Gicquel B, et al Strain identification of Mycobacterium tuberculosis by DNA fingerprinting: recommendations for a standardized methodology. Journal of Clinical Microbiology. 1993;31(2):406 8381814 10.1128/jcm.31.2.406-409.1993 PMC262774

[15] Gagneux S, Small PM. Global phylogeography of Mycobacterium tuberculosis and implications for tuberculosis product development. The Lancet Infectious Diseases. 2007;7(5):328–37. 10.1016/S1473-3099(07)70108-1 17448936

[16] Meehan CJ, Moris P, Kohl TA, Pecerska J, Akter S, Merker M, et al The relationship between transmission time and clustering methods in Mycobacterium tuberculosis epidemiology. EBioMedicine. 2018;37(2018):410–16. Epub 16 October 2018. https://dx.doi.org/10.1016%2Fj.ebiom.2018.10.013. PubMed Central PMCID: PMChttps://www.ncbi.nlm.nih.gov/pubmed/30341041. 30341041 10.1016/j.ebiom.2018.10.013 PMC6284411

[17] Maguga-Phasha NTC, Munyai NS, Mashinya F, Makgatho ME, Mbajiorgu EF. Genetic diversity and distribution of Mycobacterium tuberculosis genotypes in Limpopo, South Africa. BMC Infect Dis. 2017;17(1):764 10.1186/s12879-017-2881-z 29233106 PMC5727936

[18] Lockman S, Sheppard JD, Braden CR, Mwasekaga MJ, Woodley CL, Kenyon TA, et al Molecular and conventional epidemiology of Mycobacterium tuberculosis in Botswana: a population-based prospective study of 301 pulmonary tuberculosis patients. J Clin Microbiol. 2001;39(3):1042–7. Epub 2001/03/07. 10.1128/JCM.39.3.1042-1047.2001 11230425 PMC87871

[19] Lockman S, Sheppard JD, Mwasekaga M, Kenyon TA, Binkin NJ, Braden CR, et al DNA fingerprinting of a national sample of Mycobacterium tuberculosis isolates, Botswana, 1995–1996. Int J Tuberc Lung Dis. 2000;4(6):584–7. Epub 2000/06/23. . 10864192

[20] Supply P, Allix C, Lesjean S, Cardoso-Oelemann M, Rusch-Gerdes S, Willery E, et al Proposal for standardization of optimized mycobacterial interspersed repetitive unit-variable-number tandem repeat typing of Mycobacterium tuberculosis. J Clin Microbiol. 2006;44(12):4498–510. Epub 2006/09/29. 10.1128/JCM.01392-06 17005759 PMC1698431

[21] Chihota VN, Niehaus A, Streicher EM, Wang X, Sampson SL, Mason P, et al Geospatial distribution of Mycobacterium tuberculosis genotypes in Africa. PLOS ONE. 2018;13(8):e0200632 10.1371/journal.pone.0200632 30067763 PMC6070189

[22] Mbugi EV, Katale BZ, Streicher EM, Keyyu JD, Kendall SL, Dockrell HM, et al Mapping of Mycobacterium tuberculosis Complex Genetic Diversity Profiles in Tanzania and Other African Countries. PloS one. 2016;11(5):e0154571 Epub 2016/05/07. 10.1371/journal.pone.0154571 27149626 PMC4858144

[23] Viegas SO, Machado A Fau—Groenheit R, Groenheit R Fau—Ghebremichael S, Ghebremichael S Fau—Pennhag A, Pennhag A Fau—Gudo PS, Gudo Ps Fau—Cuna Z, et al Molecular diversity of Mycobacterium tuberculosis isolates from patients with pulmonary tuberculosis in Mozambique. BMC Microbiol. 2010;10(195). Epub 2010 Jul 21. 10.1186/1471-2180-10-195 20663126 PMC2914001

[24] de Jong BC, Antonio M, Awine T, Ogungbemi K, de Jong YP, Gagneux S, et al Use of spoligotyping and large sequence polymorphisms to study the population structure of the Mycobacterium tuberculosis complex in a cohort study of consecutive smear-positive tuberculosis cases in The Gambia. J Clin Microbiol. 2009;47(4):994–1001. Epub 2009/02/06. 10.1128/JCM.01216-08 19193842 PMC2668362

[25] Gehre F, Kumar S, Kendall L, Ejo M, Secka O, Ofori-Anyinam B, et al A Mycobacterial Perspective on Tuberculosis in West Africa: Significant Geographical Variation of M. africanum and Other M. tuberculosis Complex Lineages. PLOS Neglected Tropical Diseases. 2016;10(3):e0004408 10.1371/journal.pntd.0004408 26964059 PMC4786107

[26] Koivula T, Ekman M Fau—Leitner T, Leitner T Fau—Lofdahl S, Lofdahl S Fau—Ghebremicahel S, Ghebremicahel S Fau—Mostowy S, Mostowy S Fau—Behr MA, et al Genetic characterization of the Guinea-Bissau family of Mycobacterium tuberculosis complex strains. Microbes and Infection. 2004;6(3):272–8. 10.1016/j.micinf.2003.12.006 15026014

[27] Jori F M. M, Etter E, Munstermann S, Newman SH, Michel A. Preliminary assessment of bovine tuberculosis at the livestock/wildlife interface in two protected areas of northern Botswana. Transboundary and Emerging Diseases. 2013;60(s1):28–36. 10.1111/tbed.12110. 24171846

[28] Lahlou O, Millet J, Chaoui I, Sabouni R, Filali-Maltouf A, Akrim M, et al The Genotypic Population Structure of Mycobacterium tuberculosis Complex from Moroccan Patients Reveals a Predominance of Euro-American Lineages. PLOS ONE. 2012;7(10):e47113 10.1371/journal.pone.0047113 23077552 PMC3471964

[29] Menzies HJ, Moalosi G, Anisimova V, Gammino V, Sentle C, Bachhuber MA, et al Increase in anti-tuberculosis drug resistance in Botswana: results from the fourth National Drug Resistance Survey. Int J Tuberc Lung Dis. 2014;18(9):1026–33. Epub 2014/09/06. 10.5588/ijtld.13.0749 25189548 PMC5531285

[30] 4th National Anti-tuberculosis Drug Resistance Survey (DRS) Botswana, 2007–2008. 2010 November 2010. Report No.

[31] Mesfin EA, Beyene D, Tesfaye A, Admasu A, Addise D, Amare M, et al Drug-resistance patterns of Mycobacterium tuberculosis strains and associated risk factors among multi drug-resistant tuberculosis suspected patients from Ethiopia. PLoS One. 2018;13(6):e0197737 Epub 2018/06/05. 10.1371/journal.pone.0197737 29864118 PMC5986145

[32] Cox H, Kebede Y, Allamuratova S, Ismailov G, Davletmuratova Z, Byrnes G, et al Tuberculosis Recurrence and Mortality after Successful Treatment: Impact of Drug Resistance. PLOS Medicine. 2006;3(10):e384 10.1371/journal.pmed.0030384 17020405 PMC1584414

[33] Munang ML, Kariuki M, Dedicoat M. Isoniazid-resistant tuberculosis in Birmingham, United Kingdom, 1999–2010. QJM: An International Journal of Medicine. 2015;108(1):19–25. 24989780 10.1093/qjmed/hcu139

[34] Kibiki GS, Mulder B, Dolmans WM, de Beer JL, Boeree M, Sam N, et al M. tuberculosis genotypic diversity and drug susceptibility pattern in HIV-infected and non-HIV-infected patients in northern Tanzania. BMC Microbiol. 2007;7(51). Epub 2007/06/02. 10.1186/1471-2180-7-51 17540031 PMC1913919

[35] Nelson KN, Shah NS, Mathema B, Ismail N, Brust JCM, Brown TS, et al Spatial Patterns of Extensively Drug-Resistant Tuberculosis Transmission in KwaZulu-Natal, South Africa. J Infect Dis. 2018;218(12):1964–73. Epub 2018/07/03. 10.1093/infdis/jiy394 29961879 PMC6217717

[36] Auld SC, Shah NS, Mathema B, Brown TS, Ismail N, Omar SV, et al Extensively drug-resistant tuberculosis in South Africa: genomic evidence supporting transmission in communities. Eur Respir J. 2018;52(4). Epub 2018/08/18. 10.1183/13993003.00246-2018 . 30115614 PMC6195447

