# Supplementary material for: Genetic diversity of Mycobacterium tuberculosis strains circulating in Botswana
Source: PLoS One. 2019 May 7;14(5):e0216306. doi: 10.1371/journal.pone.0216306 (PMC6504092; doi:10.1371/journal.pone.0216306)
Supplement: S1 Table — (DOCX) [file pone.0216306.s001.docx]

**S1 Table 1: Spoligotype International types (SIT) and corresponding spoligotyping-defined families and lineages of *M. tuberculosis* isolates included in the study**

| SIT | Octal code | Spoligotype pattern | Family | Lineage | Frequency | | Unique/Clustered | |
| --- | --- | --- | --- | --- | --- | --- | --- | --- |
| ORPHAN | 700076770000071 | ⬛⬛⬛⬜⬜⬜⬜⬜⬜⬜⬜⬜⬛⬛⬛⬛⬛⬜⬛⬛⬛⬛⬛⬛⬜⬜⬜⬜⬜⬜⬜⬜⬜⬜⬜⬜⬜⬜⬜⬛⬛⬛⬛ | Afri | Lineage 6 | 1 | Unique | |  |
| 1 | 000000000003771 | ⬜⬜⬜⬜⬜⬜⬜⬜⬜⬜⬜⬜⬜⬜⬜⬜⬜⬜⬜⬜⬜⬜⬜⬜⬜⬜⬜⬜⬜⬜⬜⬜⬜⬜⬛⬛⬛⬛⬛⬛⬛⬛⬛ | Beijing | Lineage 2 | 39 | Clustered | |  |
| 190 | 000000000003731 | ⬜⬜⬜⬜⬜⬜⬜⬜⬜⬜⬜⬜⬜⬜⬜⬜⬜⬜⬜⬜⬜⬜⬜⬜⬜⬜⬜⬜⬜⬜⬜⬜⬜⬜⬛⬛⬛⬛⬛⬜⬛⬛⬛ | Beijing | Lineage 2 | 2 | Clustered | |  |
| 683 | 664073777777600 | ⬛⬛⬜⬛⬛⬜⬛⬜⬜⬜⬜⬜⬛⬛⬛⬜⬛⬛⬛⬛⬛⬛⬛⬛⬛⬛⬛⬛⬛⬛⬛⬛⬛⬛⬛⬛⬛⬛⬜⬜⬜⬜⬜ | BOVIS2 | Bovis | 1 | Unique | |  |
| 21 | 703377400001771 | ⬛⬛⬛⬜⬜⬜⬜⬛⬛⬜⬛⬛⬛⬛⬛⬛⬛⬛⬛⬜⬜⬜⬜⬜⬜⬜⬜⬜⬜⬜⬜⬜⬜⬜⬜⬛⬛⬛⬛⬛⬛⬛⬛ | CAS1_KILI | Lineage 1 | 2 | Clustered | |  |
| 129 | 700777747413771 | ⬛⬛⬛⬜⬜⬜⬜⬜⬜⬛⬛⬛⬛⬛⬛⬛⬛⬛⬛⬛⬛⬛⬜⬜⬛⬛⬛⬛⬜⬜⬜⬜⬛⬜⬛⬛⬛⬛⬛⬛⬛⬛⬛ | EAI5 | Lineage 1 | 4 | Clustered | |  |
| 236 | 777777777413771 | ⬛⬛⬛⬛⬛⬛⬛⬛⬛⬛⬛⬛⬛⬛⬛⬛⬛⬛⬛⬛⬛⬛⬛⬛⬛⬛⬛⬛⬜⬜⬜⬜⬛⬜⬛⬛⬛⬛⬛⬛⬛⬛⬛ | EAI5 | Lineage 1 | 1 | Unique | |  |
| 625 | 477777777412071 | ⬛⬜⬜⬛⬛⬛⬛⬛⬛⬛⬛⬛⬛⬛⬛⬛⬛⬛⬛⬛⬛⬛⬛⬛⬛⬛⬛⬛⬜⬜⬜⬜⬛⬜⬛⬜⬜⬜⬜⬛⬛⬛⬛ | EAI5 | Lineage 1 | 2 | Clustered | |  |
| 1467 | 477777777410771 | ⬛⬜⬜⬛⬛⬛⬛⬛⬛⬛⬛⬛⬛⬛⬛⬛⬛⬛⬛⬛⬛⬛⬛⬛⬛⬛⬛⬛⬜⬜⬜⬜⬛⬜⬜⬜⬛⬛⬛⬛⬛⬛⬛ | EAI5 | Lineage 1 | 1 | Unique | |  |
| 48 | 777777777413731 | ⬛⬛⬛⬛⬛⬛⬛⬛⬛⬛⬛⬛⬛⬛⬛⬛⬛⬛⬛⬛⬛⬛⬛⬛⬛⬛⬛⬛⬜⬜⬜⬜⬛⬜⬛⬛⬛⬛⬛⬜⬛⬛⬛ | EAI1_SOM | Lineage 1 | 13 | Clustered | |  |
| 337 | 777777777013731 | ⬛⬛⬛⬛⬛⬛⬛⬛⬛⬛⬛⬛⬛⬛⬛⬛⬛⬛⬛⬛⬛⬛⬛⬛⬛⬛⬛⬜⬜⬜⬜⬜⬛⬜⬛⬛⬛⬛⬛⬜⬛⬛⬛ | EAI1_SOM | Lineage 1 | 1 | Unique | |  |
| 711 | 737777777413731 | ⬛⬛⬛⬜⬛⬛⬛⬛⬛⬛⬛⬛⬛⬛⬛⬛⬛⬛⬛⬛⬛⬛⬛⬛⬛⬛⬛⬛⬜⬜⬜⬜⬛⬜⬛⬛⬛⬛⬛⬜⬛⬛⬛ | EAI1_SOM | Lineage 1 | 1 | Unique | |  |
| 806 | 757777777413731 | ⬛⬛⬛⬛⬜⬛⬛⬛⬛⬛⬛⬛⬛⬛⬛⬛⬛⬛⬛⬛⬛⬛⬛⬛⬛⬛⬛⬛⬜⬜⬜⬜⬛⬜⬛⬛⬛⬛⬛⬜⬛⬛⬛ | EAI1_SOM | Lineage 1 | 2 | Clustered | |  |
| 1649 | 757677777413731 | ⬛⬛⬛⬛⬜⬛⬛⬛⬛⬛⬛⬜⬛⬛⬛⬛⬛⬛⬛⬛⬛⬛⬛⬛⬛⬛⬛⬛⬜⬜⬜⬜⬛⬜⬛⬛⬛⬛⬛⬜⬛⬛⬛ | EAI1_SOM | Lineage 1 | 1 | Unique | |  |
| Unknown | 777767777413621 | ⬛⬛⬛⬛⬛⬛⬛⬛⬛⬛⬛⬛⬛⬛⬜⬛⬛⬛⬛⬛⬛⬛⬛⬛⬛⬛⬛⬛⬜⬜⬜⬜⬛⬜⬛⬛⬛⬛⬜⬜⬛⬜⬛ | EAI | Lineage 1 | 1 | Unique | |  |
| Unknown | 757777777411731 | ⬛⬛⬛⬛⬜⬛⬛⬛⬛⬛⬛⬛⬛⬛⬛⬛⬛⬛⬛⬛⬛⬛⬛⬛⬛⬛⬛⬛⬜⬜⬜⬜⬛⬜⬜⬛⬛⬛⬛⬜⬛⬛⬛ | EAI | Lineage 1 | 1 | Unique | |  |
| Unknown | 777777777410731 | ⬛⬛⬛⬛⬛⬛⬛⬛⬛⬛⬛⬛⬛⬛⬛⬛⬛⬛⬛⬛⬛⬛⬛⬛⬛⬛⬛⬛⬜⬜⬜⬜⬛⬜⬜⬜⬛⬛⬛⬜⬛⬛⬛ | EAI | Lineage 1 | 1 | Unique | |  |
| Unknown | 437777777410771 | ⬛⬜⬜⬜⬛⬛⬛⬛⬛⬛⬛⬛⬛⬛⬛⬛⬛⬛⬛⬛⬛⬛⬛⬛⬛⬛⬛⬛⬜⬜⬜⬜⬛⬜⬜⬜⬛⬛⬛⬛⬛⬛⬛ | EAI | Lineage 1 | 2 | Clustered | |  |
| 47 | 777777774020771 | ⬛⬛⬛⬛⬛⬛⬛⬛⬛⬛⬛⬛⬛⬛⬛⬛⬛⬛⬛⬛⬛⬛⬛⬛⬛⬜⬜⬜⬜⬜⬜⬛⬜⬜⬜⬜⬛⬛⬛⬛⬛⬛⬛ | H1 | Lineage 4 | 2 | Clustered | |  |
| 62 | 777777774020731 | ⬛⬛⬛⬛⬛⬛⬛⬛⬛⬛⬛⬛⬛⬛⬛⬛⬛⬛⬛⬛⬛⬛⬛⬛⬛⬜⬜⬜⬜⬜⬜⬛⬜⬜⬜⬜⬛⬛⬛⬜⬛⬛⬛ | H1 | Lineage 4 | 9 | Clustered | |  |
| 218 | 777737774020771 | ⬛⬛⬛⬛⬛⬛⬛⬛⬛⬛⬛⬛⬜⬛⬛⬛⬛⬛⬛⬛⬛⬛⬛⬛⬛⬜⬜⬜⬜⬜⬜⬛⬜⬜⬜⬜⬛⬛⬛⬛⬛⬛⬛ | H1 | Lineage 4 | 1 | Unique | |  |
| 382 | 757777774020771 | ⬛⬛⬛⬛⬜⬛⬛⬛⬛⬛⬛⬛⬛⬛⬛⬛⬛⬛⬛⬛⬛⬛⬛⬛⬛⬜⬜⬜⬜⬜⬜⬛⬜⬜⬜⬜⬛⬛⬛⬛⬛⬛⬛ | H1 | Lineage 4 | 1 | Unique | |  |
| 1988 | 777777604020731 | ⬛⬛⬛⬛⬛⬛⬛⬛⬛⬛⬛⬛⬛⬛⬛⬛⬛⬛⬛⬛⬜⬜⬜⬜⬛⬜⬜⬜⬜⬜⬜⬛⬜⬜⬜⬜⬛⬛⬛⬜⬛⬛⬛ | H1 | Lineage 4 | 1 | Unique | |  |
| Unknown | 777677774020731 | ⬛⬛⬛⬛⬛⬛⬛⬛⬛⬛⬛⬜⬛⬛⬛⬛⬛⬛⬛⬛⬛⬛⬛⬛⬛⬜⬜⬜⬜⬜⬜⬛⬜⬜⬜⬜⬛⬛⬛⬜⬛⬛⬛ | H1 | Lineage 4 | 2 | Clustered | |  |
| Unknown | 777777774020531 | ⬛⬛⬛⬛⬛⬛⬛⬛⬛⬛⬛⬛⬛⬛⬛⬛⬛⬛⬛⬛⬛⬛⬛⬛⬛⬜⬜⬜⬜⬜⬜⬛⬜⬜⬜⬜⬛⬜⬛⬜⬛⬛⬛ | H1 | Lineage 4 | 1 | Unique | |  |
| 36 | 777737777720771 | ⬛⬛⬛⬛⬛⬛⬛⬛⬛⬛⬛⬛⬜⬛⬛⬛⬛⬛⬛⬛⬛⬛⬛⬛⬛⬛⬛⬛⬛⬛⬜⬛⬜⬜⬜⬜⬛⬛⬛⬛⬛⬛⬛ | H3 | Lineage 4 | 1 | Unique | |  |
| 50 | 777777777720771 | ⬛⬛⬛⬛⬛⬛⬛⬛⬛⬛⬛⬛⬛⬛⬛⬛⬛⬛⬛⬛⬛⬛⬛⬛⬛⬛⬛⬛⬛⬛⬜⬛⬜⬜⬜⬜⬛⬛⬛⬛⬛⬛⬛ | H3 | Lineage 4 | 1 | Unique | |  |
| 946 | 777777740020771 | ⬛⬛⬛⬛⬛⬛⬛⬛⬛⬛⬛⬛⬛⬛⬛⬛⬛⬛⬛⬛⬛⬛⬜⬜⬜⬜⬜⬜⬜⬜⬜⬛⬜⬜⬜⬜⬛⬛⬛⬛⬛⬛⬛ | H3 | Lineage 4 | 1 | Unique | |  |
| ORPHAN | 777777740020731 | ⬛⬛⬛⬛⬛⬛⬛⬛⬛⬛⬛⬛⬛⬛⬛⬛⬛⬛⬛⬛⬛⬛⬜⬜⬜⬜⬜⬜⬜⬜⬜⬛⬜⬜⬜⬜⬛⬛⬛⬜⬛⬛⬛ | H3 | Lineage 4 | 1 | Unique | |  |
| 451 | 777777477760771 | ⬛⬛⬛⬛⬛⬛⬛⬛⬛⬛⬛⬛⬛⬛⬛⬛⬛⬛⬛⬜⬜⬛⬛⬛⬛⬛⬛⬛⬛⬛⬛⬛⬜⬜⬜⬜⬛⬛⬛⬛⬛⬛⬛ | H37RV | Lineage 4 | 2 | Clustered | |  |
| 4 | 000000007760771 | ⬜⬜⬜⬜⬜⬜⬜⬜⬜⬜⬜⬜⬜⬜⬜⬜⬜⬜⬜⬜⬜⬜⬜⬜⬛⬛⬛⬛⬛⬛⬛⬛⬜⬜⬜⬜⬛⬛⬛⬛⬛⬛⬛ | LAM3/S convergent | Lineage 4 | 1 | Unique | |  |
| 20 | 677777607760771 | ⬛⬛⬜⬛⬛⬛⬛⬛⬛⬛⬛⬛⬛⬛⬛⬛⬛⬛⬛⬛⬜⬜⬜⬜⬛⬛⬛⬛⬛⬛⬛⬛⬜⬜⬜⬜⬛⬛⬛⬛⬛⬛⬛ | LAM1 | Lineage 4 | 14 | Clustered | |  |
| 1713 | 663777607760771 | ⬛⬛⬜⬛⬛⬜⬜⬛⬛⬛⬛⬛⬛⬛⬛⬛⬛⬛⬛⬛⬜⬜⬜⬜⬛⬛⬛⬛⬛⬛⬛⬛⬜⬜⬜⬜⬛⬛⬛⬛⬛⬛⬛ | LAM1 | Lineage 4 | 1 | Unique | |  |
| 2342 | 037777607760771 | ⬜⬜⬜⬜⬛⬛⬛⬛⬛⬛⬛⬛⬛⬛⬛⬛⬛⬛⬛⬛⬜⬜⬜⬜⬛⬛⬛⬛⬛⬛⬛⬛⬜⬜⬜⬜⬛⬛⬛⬛⬛⬛⬛ | LAM1 | Lineage 4 | 1 | Unique | |  |
| Unknown | 677347607760661 | ⬛⬛⬜⬛⬛⬛⬛⬛⬛⬜⬛⬛⬛⬜⬜⬛⬛⬛⬛⬛⬜⬜⬜⬜⬛⬛⬛⬛⬛⬛⬛⬛⬜⬜⬜⬜⬛⬛⬜⬛⬛⬜⬛ | LAM1 | Lineage 4 | 1 | Unique | |  |
| 1321 | 677777607760731 | ⬛⬛⬜⬛⬛⬛⬛⬛⬛⬛⬛⬛⬛⬛⬛⬛⬛⬛⬛⬛⬜⬜⬜⬜⬛⬛⬛⬛⬛⬛⬛⬛⬜⬜⬜⬜⬛⬛⬛⬜⬛⬛⬛ | LAM1-LAM4 | Lineage 4 | 1 | Unique | |  |
| 33 | 776177607760771 | ⬛⬛⬛⬛⬛⬛⬛⬛⬜⬜⬜⬛⬛⬛⬛⬛⬛⬛⬛⬛⬜⬜⬜⬜⬛⬛⬛⬛⬛⬛⬛⬛⬜⬜⬜⬜⬛⬛⬛⬛⬛⬛⬛ | LAM3 | Lineage 4 | 25 | Clustered | |  |
| 130 | 776177607760731 | ⬛⬛⬛⬛⬛⬛⬛⬛⬜⬜⬜⬛⬛⬛⬛⬛⬛⬛⬛⬛⬜⬜⬜⬜⬛⬛⬛⬛⬛⬛⬛⬛⬜⬜⬜⬜⬛⬛⬛⬜⬛⬛⬛ | LAM3 | Lineage 4 | 1 | Unique | |  |
| 719 | 776177407760771 | ⬛⬛⬛⬛⬛⬛⬛⬛⬜⬜⬜⬛⬛⬛⬛⬛⬛⬛⬛⬜⬜⬜⬜⬜⬛⬛⬛⬛⬛⬛⬛⬛⬜⬜⬜⬜⬛⬛⬛⬛⬛⬛⬛ | LAM3 | Lineage 4 | 14 | Clustered | |  |
| 1293 | 676177607760771 | ⬛⬛⬜⬛⬛⬛⬛⬛⬜⬜⬜⬛⬛⬛⬛⬛⬛⬛⬛⬛⬜⬜⬜⬜⬛⬛⬛⬛⬛⬛⬛⬛⬜⬜⬜⬜⬛⬛⬛⬛⬛⬛⬛ | LAM3 | Lineage 4 | 1 | Unique | |  |
| ORPHAN | 776167407760771 | ⬛⬛⬛⬛⬛⬛⬛⬛⬜⬜⬜⬛⬛⬛⬜⬛⬛⬛⬛⬜⬜⬜⬜⬜⬛⬛⬛⬛⬛⬛⬛⬛⬜⬜⬜⬜⬛⬛⬛⬛⬛⬛⬛ | LAM3 | Lineage 4 | 1 | Unique | |  |
| Unknown | 774000007760731 | ⬛⬛⬛⬛⬛⬛⬛⬜⬜⬜⬜⬜⬜⬜⬜⬜⬜⬜⬜⬜⬜⬜⬜⬜⬛⬛⬛⬛⬛⬛⬛⬛⬜⬜⬜⬜⬛⬛⬛⬜⬛⬛⬛ | LAM3 | Lineage 4 | 2 | Clustered | |  |
| Unknown | 774037607760700 | ⬛⬛⬛⬛⬛⬛⬛⬜⬜⬜⬜⬜⬜⬛⬛⬛⬛⬛⬛⬛⬜⬜⬜⬜⬛⬛⬛⬛⬛⬛⬛⬛⬜⬜⬜⬜⬛⬛⬛⬜⬜⬜⬜ | LAM3 | Lineage 4 | 1 | Unique | |  |
| 60 | 777777607760731 | ⬛⬛⬛⬛⬛⬛⬛⬛⬛⬛⬛⬛⬛⬛⬛⬛⬛⬛⬛⬛⬜⬜⬜⬜⬛⬛⬛⬛⬛⬛⬛⬛⬜⬜⬜⬜⬛⬛⬛⬜⬛⬛⬛ | LAM4 | Lineage 4 | 3 | Clustered | |  |
| 811 | 777777604060731 | ⬛⬛⬛⬛⬛⬛⬛⬛⬛⬛⬛⬛⬛⬛⬛⬛⬛⬛⬛⬛⬜⬜⬜⬜⬛⬜⬜⬜⬜⬜⬛⬛⬜⬜⬜⬜⬛⬛⬛⬜⬛⬛⬛ | LAM4 | Lineage 4 | 9 | Clustered | |  |
| 828 | 377777607760731 | ⬜⬛⬛⬛⬛⬛⬛⬛⬛⬛⬛⬛⬛⬛⬛⬛⬛⬛⬛⬛⬜⬜⬜⬜⬛⬛⬛⬛⬛⬛⬛⬛⬜⬜⬜⬜⬛⬛⬛⬜⬛⬛⬛ | LAM4 | Lineage 4 | 1 | Unique | |  |
| 42 | 777777607760771 | ⬛⬛⬛⬛⬛⬛⬛⬛⬛⬛⬛⬛⬛⬛⬛⬛⬛⬛⬛⬛⬜⬜⬜⬜⬛⬛⬛⬛⬛⬛⬛⬛⬜⬜⬜⬜⬛⬛⬛⬛⬛⬛⬛ | LAM9 | Lineage 4 | 4 | Clustered | |  |
| Unknown | 777776607760731 | ⬛⬛⬛⬛⬛⬛⬛⬛⬛⬛⬛⬛⬛⬛⬛⬛⬛⬜⬛⬛⬜⬜⬜⬜⬛⬛⬛⬛⬛⬛⬛⬛⬜⬜⬜⬜⬛⬛⬛⬜⬛⬛⬛ | LAM9 | Lineage 4 | 1 | Unique | |  |
| 59 | 777777606060771 | ⬛⬛⬛⬛⬛⬛⬛⬛⬛⬛⬛⬛⬛⬛⬛⬛⬛⬛⬛⬛⬜⬜⬜⬜⬛⬛⬜⬜⬜⬜⬛⬛⬜⬜⬜⬜⬛⬛⬛⬛⬛⬛⬛ | LAM11_ZWE | Lineage 4 | 8 | Clustered | |  |
| 813 | 777777606060631 | ⬛⬛⬛⬛⬛⬛⬛⬛⬛⬛⬛⬛⬛⬛⬛⬛⬛⬛⬛⬛⬜⬜⬜⬜⬛⬛⬜⬜⬜⬜⬛⬛⬜⬜⬜⬜⬛⬛⬜⬜⬛⬛⬛ | LAM11_ZWE | Lineage 4 | 1 | Unique | |  |
| 815 | 777777606060731 | ⬛⬛⬛⬛⬛⬛⬛⬛⬛⬛⬛⬛⬛⬛⬛⬛⬛⬛⬛⬛⬜⬜⬜⬜⬛⬛⬜⬜⬜⬜⬛⬛⬜⬜⬜⬜⬛⬛⬛⬜⬛⬛⬛ | LAM11_ZWE | Lineage 4 | 33 | Clustered | |  |
| 816 | 077777606060771 | ⬜⬜⬜⬛⬛⬛⬛⬛⬛⬛⬛⬛⬛⬛⬛⬛⬛⬛⬛⬛⬜⬜⬜⬜⬛⬛⬜⬜⬜⬜⬛⬛⬜⬜⬜⬜⬛⬛⬛⬛⬛⬛⬛ | LAM11_ZWE | Lineage 4 | 1 | Unique | |  |
| 1873 | 777774606060731 | ⬛⬛⬛⬛⬛⬛⬛⬛⬛⬛⬛⬛⬛⬛⬛⬛⬜⬜⬛⬛⬜⬜⬜⬜⬛⬛⬜⬜⬜⬜⬛⬛⬜⬜⬜⬜⬛⬛⬛⬜⬛⬛⬛ | LAM11_ZWE | Lineage 4 | 1 | Unique | |  |
| 2196 | 777775606060731 | ⬛⬛⬛⬛⬛⬛⬛⬛⬛⬛⬛⬛⬛⬛⬛⬛⬜⬛⬛⬛⬜⬜⬜⬜⬛⬛⬜⬜⬜⬜⬛⬛⬜⬜⬜⬜⬛⬛⬛⬜⬛⬛⬛ | LAM11_ZWE | Lineage 4 | 6 | Clustered | |  |
| ORPHAN | 777737606060731 | ⬛⬛⬛⬛⬛⬛⬛⬛⬛⬛⬛⬛⬜⬛⬛⬛⬛⬛⬛⬛⬜⬜⬜⬜⬛⬛⬜⬜⬜⬜⬛⬛⬜⬜⬜⬜⬛⬛⬛⬜⬛⬛⬛ | LAM11_ZWE | Lineage 4 | 2 | Clustered | |  |
| ORPHAN | 777777206060731 | ⬛⬛⬛⬛⬛⬛⬛⬛⬛⬛⬛⬛⬛⬛⬛⬛⬛⬛⬜⬛⬜⬜⬜⬜⬛⬛⬜⬜⬜⬜⬛⬛⬜⬜⬜⬜⬛⬛⬛⬜⬛⬛⬛ | LAM11_ZWE | Lineage 4 | 7 | Clustered | |  |
| Unknown | 770077006060731 | ⬛⬛⬛⬛⬛⬛⬜⬜⬜⬜⬜⬜⬛⬛⬛⬛⬛⬛⬜⬜⬜⬜⬜⬜⬛⬛⬜⬜⬜⬜⬛⬛⬜⬜⬜⬜⬛⬛⬛⬜⬛⬛⬛ | LAM11_ZWE | Lineage 4 | 1 | Unique | |  |
| Unknown | 777737606020771 | ⬛⬛⬛⬛⬛⬛⬛⬛⬛⬛⬛⬛⬜⬛⬛⬛⬛⬛⬛⬛⬜⬜⬜⬜⬛⬛⬜⬜⬜⬜⬜⬛⬜⬜⬜⬜⬛⬛⬛⬛⬛⬛⬛ | LAM11_ZWE | Lineage 4 | 1 | Unique | |  |
| Unknown | 777703604060731 | ⬛⬛⬛⬛⬛⬛⬛⬛⬛⬛⬛⬛⬜⬜⬜⬜⬛⬛⬛⬛⬜⬜⬜⬜⬛⬜⬜⬜⬜⬜⬛⬛⬜⬜⬜⬜⬛⬛⬛⬜⬛⬛⬛ | LAM11_ZWE | Lineage 4 | 1 | Unique | |  |
| Unknown | 777776606060731 | ⬛⬛⬛⬛⬛⬛⬛⬛⬛⬛⬛⬛⬛⬛⬛⬛⬛⬜⬛⬛⬜⬜⬜⬜⬛⬛⬜⬜⬜⬜⬛⬛⬜⬜⬜⬜⬛⬛⬛⬜⬛⬛⬛ | LAM11_ZWE | Lineage 4 | 1 | Unique | |  |
| Unknown | 777674606060731 | ⬛⬛⬛⬛⬛⬛⬛⬛⬛⬛⬛⬜⬛⬛⬛⬛⬜⬜⬛⬛⬜⬜⬜⬜⬛⬛⬜⬜⬜⬜⬛⬛⬜⬜⬜⬜⬛⬛⬛⬜⬛⬛⬛ | LAM11_ZWE | Lineage 4 | 1 | Unique | |  |
| Unknown | 577777604060731 | ⬛⬜⬛⬛⬛⬛⬛⬛⬛⬛⬛⬛⬛⬛⬛⬛⬛⬛⬛⬛⬜⬜⬜⬜⬛⬜⬜⬜⬜⬜⬛⬛⬜⬜⬜⬜⬛⬛⬛⬜⬛⬛⬛ | LAM11_ZWE | Lineage 4 | 1 | Unique | |  |
| Unknown | 770077006063731 | ⬛⬛⬛⬛⬛⬛⬜⬜⬜⬜⬜⬜⬛⬛⬛⬛⬛⬛⬜⬜⬜⬜⬜⬜⬛⬛⬜⬜⬜⬜⬛⬛⬜⬜⬛⬛⬛⬛⬛⬜⬛⬛⬛ | LAM11_ZWE | Lineage 4 | 1 | Unique | |  |
| Unknown | 701777206060731 | ⬛⬛⬛⬜⬜⬜⬜⬜⬛⬛⬛⬛⬛⬛⬛⬛⬛⬛⬜⬛⬜⬜⬜⬜⬛⬛⬜⬜⬜⬜⬛⬛⬜⬜⬜⬜⬛⬛⬛⬜⬛⬛⬛ | LAM11_ZWE | Lineage 4 | 1 | Unique | |  |
| Unknown | 777777604040131 | ⬛⬛⬛⬛⬛⬛⬛⬛⬛⬛⬛⬛⬛⬛⬛⬛⬛⬛⬛⬛⬜⬜⬜⬜⬛⬜⬜⬜⬜⬜⬛⬜⬜⬜⬜⬜⬜⬜⬛⬜⬛⬛⬛ | LAM11_ZWE | Lineage 4 | 1 | Unique | |  |
| 100 | 777777777773771 | ⬛⬛⬛⬛⬛⬛⬛⬛⬛⬛⬛⬛⬛⬛⬛⬛⬛⬛⬛⬛⬛⬛⬛⬛⬛⬛⬛⬛⬛⬛⬛⬛⬛⬜⬛⬛⬛⬛⬛⬛⬛⬛⬛ | MANU1 | Lineage 1 | 1 | Unique | |  |
| 1378 | 777777777770771 | ⬛⬛⬛⬛⬛⬛⬛⬛⬛⬛⬛⬛⬛⬛⬛⬛⬛⬛⬛⬛⬛⬛⬛⬛⬛⬛⬛⬛⬛⬛⬛⬛⬛⬜⬜⬜⬛⬛⬛⬛⬛⬛⬛ | MANU3 | Lineage 1 | 1 | Unique | |  |
| 34 | 776377777760771 | ⬛⬛⬛⬛⬛⬛⬛⬛⬜⬜⬛⬛⬛⬛⬛⬛⬛⬛⬛⬛⬛⬛⬛⬛⬛⬛⬛⬛⬛⬛⬛⬛⬜⬜⬜⬜⬛⬛⬛⬛⬛⬛⬛ | S | Lineage 4 | 46 | Clustered | |  |
| 71 | 776337777760771 | ⬛⬛⬛⬛⬛⬛⬛⬛⬜⬜⬛⬛⬜⬛⬛⬛⬛⬛⬛⬛⬛⬛⬛⬛⬛⬛⬛⬛⬛⬛⬛⬛⬜⬜⬜⬜⬛⬛⬛⬛⬛⬛⬛ | S | Lineage 4 | 4 | Clustered | |  |
| 107 | 036377777760771 | ⬜⬜⬜⬜⬛⬛⬛⬛⬜⬜⬛⬛⬛⬛⬛⬛⬛⬛⬛⬛⬛⬛⬛⬛⬛⬛⬛⬛⬛⬛⬛⬛⬜⬜⬜⬜⬛⬛⬛⬛⬛⬛⬛ | S | Lineage 4 | 1 | Unique | |  |
| 790 | 776377777740771 | ⬛⬛⬛⬛⬛⬛⬛⬛⬜⬜⬛⬛⬛⬛⬛⬛⬛⬛⬛⬛⬛⬛⬛⬛⬛⬛⬛⬛⬛⬛⬛⬜⬜⬜⬜⬜⬛⬛⬛⬛⬛⬛⬛ | S | Lineage 4 | 4 | Clustered | |  |
| 1938 | 776377777760770 | ⬛⬛⬛⬛⬛⬛⬛⬛⬜⬜⬛⬛⬛⬛⬛⬛⬛⬛⬛⬛⬛⬛⬛⬛⬛⬛⬛⬛⬛⬛⬛⬛⬜⬜⬜⬜⬛⬛⬛⬛⬛⬛⬜ | S | Lineage 4 | 1 | Unique | |  |
| Unknown | 776377777760761 | ⬛⬛⬛⬛⬛⬛⬛⬛⬜⬜⬛⬛⬛⬛⬛⬛⬛⬛⬛⬛⬛⬛⬛⬛⬛⬛⬛⬛⬛⬛⬛⬛⬜⬜⬜⬜⬛⬛⬛⬛⬛⬜⬛ | S | Lineage 4 | 1 | Unique | |  |
| Unknown | 740377777760711 | ⬛⬛⬛⬛⬜⬜⬜⬜⬜⬜⬛⬛⬛⬛⬛⬛⬛⬛⬛⬛⬛⬛⬛⬛⬛⬛⬛⬛⬛⬛⬛⬛⬜⬜⬜⬜⬛⬛⬛⬜⬜⬛⬛ | S | Lineage 4 | 1 | Unique | |  |
| 51 | 777777777760700 | ⬛⬛⬛⬛⬛⬛⬛⬛⬛⬛⬛⬛⬛⬛⬛⬛⬛⬛⬛⬛⬛⬛⬛⬛⬛⬛⬛⬛⬛⬛⬛⬛⬜⬜⬜⬜⬛⬛⬛⬜⬜⬜⬜ | T1 | Lineage 4 | 2 | Clustered | |  |
| 53 | 777777777760771 | ⬛⬛⬛⬛⬛⬛⬛⬛⬛⬛⬛⬛⬛⬛⬛⬛⬛⬛⬛⬛⬛⬛⬛⬛⬛⬛⬛⬛⬛⬛⬛⬛⬜⬜⬜⬜⬛⬛⬛⬛⬛⬛⬛ | T1 | Lineage 4 | 39 | Clustered | |  |
| 154 | 757777777760771 | ⬛⬛⬛⬛⬜⬛⬛⬛⬛⬛⬛⬛⬛⬛⬛⬛⬛⬛⬛⬛⬛⬛⬛⬛⬛⬛⬛⬛⬛⬛⬛⬛⬜⬜⬜⬜⬛⬛⬛⬛⬛⬛⬛ | T1 | Lineage 4 | 2 | Clustered | |  |
| 196 | 677777777760771 | ⬛⬛⬜⬛⬛⬛⬛⬛⬛⬛⬛⬛⬛⬛⬛⬛⬛⬛⬛⬛⬛⬛⬛⬛⬛⬛⬛⬛⬛⬛⬛⬛⬜⬜⬜⬜⬛⬛⬛⬛⬛⬛⬛ | T1 | Lineage 4 | 1 | Unique | |  |
| 344 | 700077777760771 | ⬛⬛⬛⬜⬜⬜⬜⬜⬜⬜⬜⬜⬛⬛⬛⬛⬛⬛⬛⬛⬛⬛⬛⬛⬛⬛⬛⬛⬛⬛⬛⬛⬜⬜⬜⬜⬛⬛⬛⬛⬛⬛⬛ | T1 | Lineage 4 | 1 | Unique | |  |
| 358 | 717777777760771 | ⬛⬛⬛⬜⬜⬛⬛⬛⬛⬛⬛⬛⬛⬛⬛⬛⬛⬛⬛⬛⬛⬛⬛⬛⬛⬛⬛⬛⬛⬛⬛⬛⬜⬜⬜⬜⬛⬛⬛⬛⬛⬛⬛ | T1 | Lineage 4 | 2 | Clustered | |  |
| 373 | 777777767760771 | ⬛⬛⬛⬛⬛⬛⬛⬛⬛⬛⬛⬛⬛⬛⬛⬛⬛⬛⬛⬛⬛⬛⬛⬜⬛⬛⬛⬛⬛⬛⬛⬛⬜⬜⬜⬜⬛⬛⬛⬛⬛⬛⬛ | T1 | Lineage 4 | 1 | Unique | |  |
| 521 | 777777777760611 | ⬛⬛⬛⬛⬛⬛⬛⬛⬛⬛⬛⬛⬛⬛⬛⬛⬛⬛⬛⬛⬛⬛⬛⬛⬛⬛⬛⬛⬛⬛⬛⬛⬜⬜⬜⬜⬛⬛⬜⬜⬜⬛⬛ | T1 | Lineage 4 | 2 | Clustered | |  |
| 966 | 775777777760771 | ⬛⬛⬛⬛⬛⬛⬛⬜⬛⬛⬛⬛⬛⬛⬛⬛⬛⬛⬛⬛⬛⬛⬛⬛⬛⬛⬛⬛⬛⬛⬛⬛⬜⬜⬜⬜⬛⬛⬛⬛⬛⬛⬛ | T1 | Lineage 4 | 1 | Unique | |  |
| 1284 | 741703777760771 | ⬛⬛⬛⬛⬜⬜⬜⬜⬛⬛⬛⬛⬜⬜⬜⬜⬛⬛⬛⬛⬛⬛⬛⬛⬛⬛⬛⬛⬛⬛⬛⬛⬜⬜⬜⬜⬛⬛⬛⬛⬛⬛⬛ | T1 | Lineage 4 | 1 | Unique | |  |
| ORPHAN | 617777777760771 | ⬛⬛⬜⬜⬜⬛⬛⬛⬛⬛⬛⬛⬛⬛⬛⬛⬛⬛⬛⬛⬛⬛⬛⬛⬛⬛⬛⬛⬛⬛⬛⬛⬜⬜⬜⬜⬛⬛⬛⬛⬛⬛⬛ | T1 | Lineage 4 | 1 | Unique | |  |
| 118 | 777767777760771 | ⬛⬛⬛⬛⬛⬛⬛⬛⬛⬛⬛⬛⬛⬛⬜⬛⬛⬛⬛⬛⬛⬛⬛⬛⬛⬛⬛⬛⬛⬛⬛⬛⬜⬜⬜⬜⬛⬛⬛⬛⬛⬛⬛ | T2 | Lineage 4 | 2 | Clustered | |  |
| 2867 | 777777775760731 | ⬛⬛⬛⬛⬛⬛⬛⬛⬛⬛⬛⬛⬛⬛⬛⬛⬛⬛⬛⬛⬛⬛⬛⬛⬛⬜⬛⬛⬛⬛⬛⬛⬜⬜⬜⬜⬛⬛⬛⬜⬛⬛⬛ | T2 | Lineage 4 | 1 | Unique | |  |
| 784 | 776377777760731 | ⬛⬛⬛⬛⬛⬛⬛⬛⬜⬜⬛⬛⬛⬛⬛⬛⬛⬛⬛⬛⬛⬛⬛⬛⬛⬛⬛⬛⬛⬛⬛⬛⬜⬜⬜⬜⬛⬛⬛⬜⬛⬛⬛ | T2-S | Lineage 4 | 1 | Unique | |  |
| 73 | 777737777760731 | ⬛⬛⬛⬛⬛⬛⬛⬛⬛⬛⬛⬛⬜⬛⬛⬛⬛⬛⬛⬛⬛⬛⬛⬛⬛⬛⬛⬛⬛⬛⬛⬛⬜⬜⬜⬜⬛⬛⬛⬜⬛⬛⬛ | T2-T3 | Lineage 4 | 5 | Clustered | |  |
| 37 | 777737777760771 | ⬛⬛⬛⬛⬛⬛⬛⬛⬛⬛⬛⬛⬜⬛⬛⬛⬛⬛⬛⬛⬛⬛⬛⬛⬛⬛⬛⬛⬛⬛⬛⬛⬜⬜⬜⬜⬛⬛⬛⬛⬛⬛⬛ | T3 | Lineage 4 | 4 | Clustered | |  |
| 158 | 777737777760601 | ⬛⬛⬛⬛⬛⬛⬛⬛⬛⬛⬛⬛⬜⬛⬛⬛⬛⬛⬛⬛⬛⬛⬛⬛⬛⬛⬛⬛⬛⬛⬛⬛⬜⬜⬜⬜⬛⬛⬜⬜⬜⬜⬛ | T3 | Lineage 4 | 1 | Unique | |  |
| 44 | 777777757760771 | ⬛⬛⬛⬛⬛⬛⬛⬛⬛⬛⬛⬛⬛⬛⬛⬛⬛⬛⬛⬛⬛⬛⬜⬛⬛⬛⬛⬛⬛⬛⬛⬛⬜⬜⬜⬜⬛⬛⬛⬛⬛⬛⬛ | T5 | Lineage 4 | 1 | Unique | |  |
| 68 | 775777757760771 | ⬛⬛⬛⬛⬛⬛⬛⬜⬛⬛⬛⬛⬛⬛⬛⬛⬛⬛⬛⬛⬛⬛⬜⬛⬛⬛⬛⬛⬛⬛⬛⬛⬜⬜⬜⬜⬛⬛⬛⬛⬛⬛⬛ | T5 | Lineage 4 | 2 | Clustered | |  |
| 46 | 777777770000000 | ⬛⬛⬛⬛⬛⬛⬛⬛⬛⬛⬛⬛⬛⬛⬛⬛⬛⬛⬛⬛⬛⬛⬛⬛⬜⬜⬜⬜⬜⬜⬜⬜⬜⬜⬜⬜⬜⬜⬜⬜⬜⬜⬜ | U (likely H) | Lineage 4 | 3 | Clustered | |  |
| 137 | 777776777760601 | ⬛⬛⬛⬛⬛⬛⬛⬛⬛⬛⬛⬛⬛⬛⬛⬛⬛⬜⬛⬛⬛⬛⬛⬛⬛⬛⬛⬛⬛⬛⬛⬛⬜⬜⬜⬜⬛⬛⬜⬜⬜⬜⬛ | X2 | Lineage 4 | 2 | Clustered | |  |
| Unknown | 617736777760601 | ⬛⬛⬜⬜⬜⬛⬛⬛⬛⬛⬛⬛⬜⬛⬛⬛⬛⬜⬛⬛⬛⬛⬛⬛⬛⬛⬛⬛⬛⬛⬛⬛⬜⬜⬜⬜⬛⬛⬜⬜⬜⬜⬛ | X2 | Lineage 4 | 1 | Unique | |  |
| 70 | 700076777760671 | ⬛⬛⬛⬜⬜⬜⬜⬜⬜⬜⬜⬜⬛⬛⬛⬛⬛⬜⬛⬛⬛⬛⬛⬛⬛⬛⬛⬛⬛⬛⬛⬛⬜⬜⬜⬜⬛⬛⬜⬛⬛⬛⬛ | X3 | Lineage 4 | 7 | Clustered | |  |
| 92 | 700076777760771 | ⬛⬛⬛⬜⬜⬜⬜⬜⬜⬜⬜⬜⬛⬛⬛⬛⬛⬜⬛⬛⬛⬛⬛⬛⬛⬛⬛⬛⬛⬛⬛⬛⬜⬜⬜⬜⬛⬛⬛⬛⬛⬛⬛ | X3 | Lineage 4 | 26 | Clustered | |  |
| 1273 | 700076777760731 | ⬛⬛⬛⬜⬜⬜⬜⬜⬜⬜⬜⬜⬛⬛⬛⬛⬛⬜⬛⬛⬛⬛⬛⬛⬛⬛⬛⬛⬛⬛⬛⬛⬜⬜⬜⬜⬛⬛⬛⬜⬛⬛⬛ | X3 | Lineage 4 | 1 | Unique | |  |
| Unknown | 400006777760771 | ⬛⬜⬜⬜⬜⬜⬜⬜⬜⬜⬜⬜⬜⬜⬜⬛⬛⬜⬛⬛⬛⬛⬛⬛⬛⬛⬛⬛⬛⬛⬛⬛⬜⬜⬜⬜⬛⬛⬛⬛⬛⬛⬛ | X3 | Lineage 4 | 1 | Unique | |  |
| Unknown | 640036777760771 | ⬛⬛⬜⬛⬜⬜⬜⬜⬜⬜⬜⬜⬜⬛⬛⬛⬛⬜⬛⬛⬛⬛⬛⬛⬛⬛⬛⬛⬛⬛⬛⬛⬜⬜⬜⬜⬛⬛⬛⬛⬛⬛⬛ | X3 | Lineage 4 | 1 | Unique | |  |
| Unknown | 400036777760771 | ⬛⬜⬜⬜⬜⬜⬜⬜⬜⬜⬜⬜⬜⬛⬛⬛⬛⬜⬛⬛⬛⬛⬛⬛⬛⬛⬛⬛⬛⬛⬛⬛⬜⬜⬜⬜⬛⬛⬛⬛⬛⬛⬛ | X3 | Lineage 4 | 30 | Clustered | |  |
| Unknown | 400076777760771 | ⬛⬜⬜⬜⬜⬜⬜⬜⬜⬜⬜⬜⬛⬛⬛⬛⬛⬜⬛⬛⬛⬛⬛⬛⬛⬛⬛⬛⬛⬛⬛⬛⬜⬜⬜⬜⬛⬛⬛⬛⬛⬛⬛ | X3 | Lineage 4 | 2 | Unique | |  |
| Unknown | 370777777760771 | ⬜⬛⬛⬛⬛⬛⬜⬜⬜⬛⬛⬛⬛⬛⬛⬛⬛⬛⬛⬛⬛⬛⬛⬛⬛⬛⬛⬛⬛⬛⬛⬛⬜⬜⬜⬜⬛⬛⬛⬛⬛⬛⬛ | Unknown | Unknown | 1 | Unique | |  |
| Unknown | 636377777760771 | ⬛⬛⬜⬜⬛⬛⬛⬛⬜⬜⬛⬛⬛⬛⬛⬛⬛⬛⬛⬛⬛⬛⬛⬛⬛⬛⬛⬛⬛⬛⬛⬛⬜⬜⬜⬜⬛⬛⬛⬛⬛⬛⬛ | Unknown | Unknown | 1 | Unique | |  |
| Unknown | 757777777001731 | ⬛⬛⬛⬛⬜⬛⬛⬛⬛⬛⬛⬛⬛⬛⬛⬛⬛⬛⬛⬛⬛⬛⬛⬛⬛⬛⬛⬜⬜⬜⬜⬜⬜⬜⬜⬛⬛⬛⬛⬜⬛⬛⬛ | Unknown | Unknown | 1 | Unique | |  |
| Unknown | 776337777740771 | ⬛⬛⬛⬛⬛⬛⬛⬛⬜⬜⬛⬛⬜⬛⬛⬛⬛⬛⬛⬛⬛⬛⬛⬛⬛⬛⬛⬛⬛⬛⬛⬜⬜⬜⬜⬜⬛⬛⬛⬛⬛⬛⬛ | Unknown | Unknown | 1 | Unique | |  |
| Unknown | 616337777760771 | ⬛⬛⬜⬜⬜⬛⬛⬛⬜⬜⬛⬛⬜⬛⬛⬛⬛⬛⬛⬛⬛⬛⬛⬛⬛⬛⬛⬛⬛⬛⬛⬛⬜⬜⬜⬜⬛⬛⬛⬛⬛⬛⬛ | Unknown | Unknown | 1 | Unique | |  |

**S2 Table 2: MIRU types (MIT) and corresponding spoligotyping-defined families and lineages for some of the drug resistant *M. tb* isolates in the study**
