# Supplementary material for: Genetic diversity of Mycobacterium tuberculosis strains circulating in Botswana
Source: PLoS One. 2019 May 7;14(5):e0216306. doi: 10.1371/journal.pone.0216306 (PMC6504092; doi:10.1371/journal.pone.0216306)
Supplement: S2 Table — (DOCX) [file pone.0216306.s002.docx]

**S2 Table 2: MIRU types (MIT) and corresponding spoligotyping-defined families and lineages for some of the drug resistant *M. tb* isolates in the study**

| SIT NUMBER | FAMILY | SPOLIGO PATTERN | MIRU PATTERN | Lineage | Number of isolates | Clustered/Unique |
| --- | --- | --- | --- | --- | --- | --- |
| 1 | BEIJING | ⬜⬜⬜⬜⬜⬜⬜⬜⬜⬜⬜⬜⬜⬜⬜⬜⬜⬜⬜⬜⬜⬜⬜⬜⬜⬜⬜⬜⬜⬜⬜⬜⬜⬜⬛⬛⬛⬛⬛⬛⬛⬛⬛ | 223325174433 | Lineage 2 | 6 | Clustered |
| 1 | BEIJING | ⬜⬜⬜⬜⬜⬜⬜⬜⬜⬜⬜⬜⬜⬜⬜⬜⬜⬜⬜⬜⬜⬜⬜⬜⬜⬜⬜⬜⬜⬜⬜⬜⬜⬜⬛⬛⬛⬛⬛⬛⬛⬛⬛ | 223325184433 | Lineage 2 | 2 | Clustered |
| 1 | BEIJING | ⬜⬜⬜⬜⬜⬜⬜⬜⬜⬜⬜⬜⬜⬜⬜⬜⬜⬜⬜⬜⬜⬜⬜⬜⬜⬜⬜⬜⬜⬜⬜⬜⬜⬜⬛⬛⬛⬛⬛⬛⬛⬛⬛ | 233325184433 | Lineage 2 | 1 | Unique |
| 34 | S | ⬛⬛⬛⬛⬛⬛⬛⬛⬜⬜⬛⬛⬛⬛⬛⬛⬛⬛⬛⬛⬛⬛⬛⬛⬛⬛⬛⬛⬛⬛⬛⬛⬜⬜⬜⬜⬛⬛⬛⬛⬛⬛⬛ | 223325143224 | Lineage 4 | 2 | Clustered |
| 34 | S | ⬛⬛⬛⬛⬛⬛⬛⬛⬜⬜⬛⬛⬛⬛⬛⬛⬛⬛⬛⬛⬛⬛⬛⬛⬛⬛⬛⬛⬛⬛⬛⬛⬜⬜⬜⬜⬛⬛⬛⬛⬛⬛⬛ | 223325153324 | Lineage 4 | 5 | Clustered |
| 34 | S | ⬛⬛⬛⬛⬛⬛⬛⬛⬜⬜⬛⬛⬛⬛⬛⬛⬛⬛⬛⬛⬛⬛⬛⬛⬛⬛⬛⬛⬛⬛⬛⬛⬜⬜⬜⬜⬛⬛⬛⬛⬛⬛⬛ | 223326153324 | Lineage 4 | 2 | Clustered |
| 20 | LAM1 | ⬛⬛⬜⬛⬛⬛⬛⬛⬛⬛⬛⬛⬛⬛⬛⬛⬛⬛⬛⬛⬜⬜⬜⬜⬛⬛⬛⬛⬛⬛⬛⬛⬜⬜⬜⬜⬛⬛⬛⬛⬛⬛⬛ | 223226353321 | Lineage 4 | 4 | Clustered |
| 33 | LAM3 | ⬛⬛⬛⬛⬛⬛⬛⬛⬜⬜⬜⬛⬛⬛⬛⬛⬛⬛⬛⬛⬜⬜⬜⬜⬛⬛⬛⬛⬛⬛⬛⬛⬜⬜⬜⬜⬛⬛⬛⬛⬛⬛⬛ | 224226153323 | Lineage 4 | 1 | Unique |
| 34 | S | ⬛⬛⬛⬛⬛⬛⬛⬛⬜⬜⬛⬛⬛⬛⬛⬛⬛⬛⬛⬛⬛⬛⬛⬛⬛⬛⬛⬛⬛⬛⬛⬛⬜⬜⬜⬜⬛⬛⬛⬛⬛⬛⬛ | 223325153424 | Lineage 4 | 1 | Unique |
| 34 | S | ⬛⬛⬛⬛⬛⬛⬛⬛⬜⬜⬛⬛⬛⬛⬛⬛⬛⬛⬛⬛⬛⬛⬛⬛⬛⬛⬛⬛⬛⬛⬛⬛⬜⬜⬜⬜⬛⬛⬛⬛⬛⬛⬛ | 223325153324 | Lineage 4 | 2 | Clustered |
| 34 | S | ⬛⬛⬛⬛⬛⬛⬛⬛⬜⬜⬛⬛⬛⬛⬛⬛⬛⬛⬛⬛⬛⬛⬛⬛⬛⬛⬛⬛⬛⬛⬛⬛⬜⬜⬜⬜⬛⬛⬛⬛⬛⬛⬛ | 223325143224 | Lineage 4 | 2 | Clustered |
| 34 | S | ⬛⬛⬛⬛⬛⬛⬛⬛⬜⬜⬛⬛⬛⬛⬛⬛⬛⬛⬛⬛⬛⬛⬛⬛⬛⬛⬛⬛⬛⬛⬛⬛⬜⬜⬜⬜⬛⬛⬛⬛⬛⬛⬛ | 223326153324 | Lineage 4 | 2 | Clustered |
| 48 | EAI1_SOM | ⬛⬛⬛⬛⬛⬛⬛⬛⬛⬛⬛⬛⬛⬛⬛⬛⬛⬛⬛⬛⬛⬛⬛⬛⬛⬛⬛⬛⬜⬜⬜⬜⬛⬜⬛⬛⬛⬛⬛⬜⬛⬛⬛ | 244326223513 | Lineage 4 | 6 | Clustered |
| 53 | T1 | ⬛⬛⬛⬛⬛⬛⬛⬛⬛⬛⬛⬛⬛⬛⬛⬛⬛⬛⬛⬛⬛⬛⬛⬛⬛⬛⬛⬛⬛⬛⬛⬛⬜⬜⬜⬜⬛⬛⬛⬛⬛⬛⬛ | 223326153331 | Lineage 4 | 1 | Unique |
| 70 | X3 | ⬛⬛⬛⬜⬜⬜⬜⬜⬜⬜⬜⬜⬛⬛⬛⬛⬛⬜⬛⬛⬛⬛⬛⬛⬛⬛⬛⬛⬛⬛⬛⬛⬜⬜⬜⬜⬛⬛⬜⬛⬛⬛⬛ | 224325153325 | Lineage 4 | 1 | Unique |
| 71 | S | ⬛⬛⬛⬛⬛⬛⬛⬛⬜⬜⬛⬛⬜⬛⬛⬛⬛⬛⬛⬛⬛⬛⬛⬛⬛⬛⬛⬛⬛⬛⬛⬛⬜⬜⬜⬜⬛⬛⬛⬛⬛⬛⬛ | 223325153324 | Lineage 4 | 1 | Unique |
| 73 | T2-T3 | ⬛⬛⬛⬛⬛⬛⬛⬛⬛⬛⬛⬛⬜⬛⬛⬛⬛⬛⬛⬛⬛⬛⬛⬛⬛⬛⬛⬛⬛⬛⬛⬛⬜⬜⬜⬜⬛⬛⬛⬜⬛⬛⬛ | 214315163221 | Lineage 4 | 1 | Unique |
| 92 | X3 | ⬛⬛⬛⬜⬜⬜⬜⬜⬜⬜⬜⬜⬛⬛⬛⬛⬛⬜⬛⬛⬛⬛⬛⬛⬛⬛⬛⬛⬛⬛⬛⬛⬜⬜⬜⬜⬛⬛⬛⬛⬛⬛⬛ | 224325123324 | Lineage 4 | 5 | Clustered |
| 137 | X2 | ⬛⬛⬛⬛⬛⬛⬛⬛⬛⬛⬛⬛⬛⬛⬛⬛⬛⬜⬛⬛⬛⬛⬛⬛⬛⬛⬛⬛⬛⬛⬛⬛⬜⬜⬜⬜⬛⬛⬜⬜⬜⬜⬛ | 233325143324 | Lineage 4 | 1 | Unique |
| 719 | LAM3 | ⬛⬛⬛⬛⬛⬛⬛⬛⬜⬜⬜⬛⬛⬛⬛⬛⬛⬛⬛⬜⬜⬜⬜⬜⬛⬛⬛⬛⬛⬛⬛⬛⬜⬜⬜⬜⬛⬛⬛⬛⬛⬛⬛ | 224226153323 | Lineage 4 | 1 | Unique |
| 719 | LAM3 | ⬛⬛⬛⬛⬛⬛⬛⬛⬜⬜⬜⬛⬛⬛⬛⬛⬛⬛⬛⬜⬜⬜⬜⬜⬛⬛⬛⬛⬛⬛⬛⬛⬜⬜⬜⬜⬛⬛⬛⬛⬛⬛⬛ | 224326153323 | Lineage 4 | 1 | Unique |
| 719 | LAM3 | ⬛⬛⬛⬛⬛⬛⬛⬛⬜⬜⬜⬛⬛⬛⬛⬛⬛⬛⬛⬜⬜⬜⬜⬜⬛⬛⬛⬛⬛⬛⬛⬛⬜⬜⬜⬜⬛⬛⬛⬛⬛⬛⬛ | 224326153333 | Lineage 4 | 2 | Clustered |
| 719 | LAM3 | ⬛⬛⬛⬛⬛⬛⬛⬛⬜⬜⬜⬛⬛⬛⬛⬛⬛⬛⬛⬜⬜⬜⬜⬜⬛⬛⬛⬛⬛⬛⬛⬛⬜⬜⬜⬜⬛⬛⬛⬛⬛⬛⬛ | 224326153333 | Lineage 4 | 1 | Unique |
| 811 | LAM4 | ⬛⬛⬛⬛⬛⬛⬛⬛⬛⬛⬛⬛⬛⬛⬛⬛⬛⬛⬛⬛⬜⬜⬜⬜⬛⬜⬜⬜⬜⬜⬛⬛⬜⬜⬜⬜⬛⬛⬛⬜⬛⬛⬛ | 223124152321 | Lineage 4 | 1 | Unique |
| 813 | LAM11_ZWE | ⬛⬛⬛⬛⬛⬛⬛⬛⬛⬛⬛⬛⬛⬛⬛⬛⬛⬛⬛⬛⬜⬜⬜⬜⬛⬛⬜⬜⬜⬜⬛⬛⬜⬜⬜⬜⬛⬛⬜⬜⬛⬛⬛ | 214125152221 | Lineage 4 | 1 | Unique |
| 815 | LAM11_ZWE | ⬛⬛⬛⬛⬛⬛⬛⬛⬛⬛⬛⬛⬛⬛⬛⬛⬛⬛⬛⬛⬜⬜⬜⬜⬛⬛⬜⬜⬜⬜⬛⬛⬜⬜⬜⬜⬛⬛⬛⬜⬛⬛⬛ | 212125152221 | Lineage 4 | 3 | Clustered |
| 815 | LAM11_ZWE | ⬛⬛⬛⬛⬛⬛⬛⬛⬛⬛⬛⬛⬛⬛⬛⬛⬛⬛⬛⬛⬜⬜⬜⬜⬛⬛⬜⬜⬜⬜⬛⬛⬜⬜⬜⬜⬛⬛⬛⬜⬛⬛⬛ | 224125152221 | Lineage 4 | 1 | Unique |
| Unknown | X3 | ⬛⬜⬜⬜⬜⬜⬜⬜⬜⬜⬜⬜⬜⬛⬛⬛⬛⬜⬛⬛⬛⬛⬛⬛⬛⬛⬛⬛⬛⬛⬛⬛⬜⬜⬜⬜⬛⬛⬛⬛⬛⬛⬛ | 214325153323 | Lineage 4 | 1 | Unique |
| Unknown | X3 | ⬛⬜⬜⬜⬜⬜⬜⬜⬜⬜⬜⬜⬜⬛⬛⬛⬛⬜⬛⬛⬛⬛⬛⬛⬛⬛⬛⬛⬛⬛⬛⬛⬜⬜⬜⬜⬛⬛⬛⬛⬛⬛⬛ | 224325153323 | Lineage 4 | 2 | Clustered |
| Unknown | X3 | ⬛⬜⬜⬜⬜⬜⬜⬜⬜⬜⬜⬜⬜⬛⬛⬛⬛⬜⬛⬛⬛⬛⬛⬛⬛⬛⬛⬛⬛⬛⬛⬛⬜⬜⬜⬜⬛⬛⬛⬛⬛⬛⬛ | 234325153323 | Lineage 4 | 1 | Unique |
| Unknown | EAI | ⬛⬛⬛⬛⬛⬛⬛⬛⬛⬛⬛⬛⬛⬛⬛⬛⬛⬛⬛⬛⬛⬛⬛⬛⬛⬛⬛⬛⬜⬜⬜⬜⬛⬜⬜⬜⬛⬛⬛⬜⬛⬛⬛ | 254326223513 | Lineage 1 | 1 | Unique |
